# Direct cleavage of caspase-8 by herpes simplex virus 1 tegument protein US11

**DOI:** 10.1038/s41598-022-15942-9

**Published:** 2022-07-19

**Authors:** Maria Musarra-Pizzo, Rosamaria Pennisi, Daniele Lombardo, Tania Velletri, Maria Teresa Sciortino

**Affiliations:** 1 Department of Chemical, Biological, Pharmaceutical and Environmental Sciences, University of Messina, 98168 Messina, Italy; 2 Division of Clinical and Molecular Hepatology, University Hospital ‘G. Martino’ of Messina, 98124 Messina, Italy; 3 IFOM-Cogentech Società Benefit Srl, via Adamello 16, 20139 Milan, Italy. Local Unit: Scientific and Technological Park of Sicily, 95121 Catania, Italy

**Keywords:** Microbiology, Virology

## Abstract

The HSV-1 tegument protein Us11 counteracts the antiviral defense mechanisms by precluding the host protein shutoff. Previous works demonstrated that Us11 prevents heat-and staurosporine-induced apoptosis and inhibits autophagy. Therefore, in the present study, we investigated the hypothesis that HSV-1, through Us11, could recruit caspase-8, a key enzyme regulating programmed cell death. We first show that HSV-1 promotes the accumulation of caspase-8-p18 active fragments in both semi permissive THP-1 cells and fully permissive HEp-2 cells to HSV-1 replication. Using a recombinant virus R3630 (ΔUs11/ΔUs12) and a plasmid encoding Us11-recombinant protein we have proven that Us11 promotes p18 accumulation, which does not trigger the apoptotic signaling. Additional, in an in vitro model, we demonstrated that Us11-recombinant protein induces caspase-8-p18 cleavage by physically interacting with the caspase-8 recombinant protein. Finally, we found that, during HSV-1 replication, activated-caspase-8 cleaves Atg3 protein to potentially block autophagy and support its replication.

## Introduction

Herpes simplex virus-1 (HSV-1) infects epithelial cells and establishes a lifelong latency in neuronal ganglia after primary infection. The ability of HSV-1 to replicate and reactivate from latency is due to several virulence factors through which the virus can evade the host antiviral response. The viral protein Us11, a true late (γ_2_) gene product, is a small phosphoprotein expressed lately during HSV-1 infection which binds to double-stranded RNA (dsRNA) and possesses multiple functions^1,2^. Us11 acts as an inhibitor of cellular pattern recognition receptor (PRR)-mediated pathways that lead to the shutoff of protein synthesis, thus maintaining protein translation late in infection. Due to its dsRNA scavenging activity, Us11 prevents IFN-β production and interferon-stimulated genes (ISG) transcription via direct interaction with RIG-I and MDA-5^3^. Besides, Us11 represses the 2’-to-5′ (2’-5′) oligoadenylate synthetase (OAS)^4^ and physically interacts with and affects the Protein Kinase-R (PKR)-mediated host immune responses, influencing the FADD/caspase-8 death-signaling pathways and autophagy response^5–7^. Indeed, it has been shown that Us11 protects HeLa cells from heat-and staurosporine-induced apoptosis and can inhibit autophagy through its interaction with PKR^8,9^. This is not surprising due to the fact that, in response to viral infection, host cells undergo autophagy and/or apoptosis to ensure degradation of viral factors and/or elimination of infected cells as well as to limit the release of progeny virus. Caspase-8 is recognized as the key enzyme for promoting apoptosis cell death. Besides its roles in apoptosis, caspase-8 is also implicated in other cell death processes like necroptosis and autophagy, performing switch point between autophagy and apoptosis as well as exerting a decisive role in cell-fate determination and immune response. Therefore, in the present research, we wondered whether the Us11 viral protein could be able to interact with and modulate caspase-8 activity. Caspase-8 molecules are synthesized as inactive zymogens with a long prodomain containing protein-protein interaction motifs defined as the death effector domains (DED1-DED2) and a C-terminal catalytic domain composed of a ~20kDa large subunit (p18) containing the active site cysteine, and a ~10kDa small subunit (p10) containing the substrate binding region^10^. The alternative splicing of *CASP8* mRNA generates several isoforms of pro-caspase-8. Caspase-8a (55kDa) and caspase-8b (54kDa,) which only differ for an additional 15 amino acids in the linker between the prodomain and catalytic domains of pro-caspase-8a, are the isoforms predominantly found^11^. The mechanism of caspase-8 expression and activation is tightly regulated through multiple processes that have been only partially elucidated. In the “two-step model”, proposed for caspase-8 autoproteolytic processing, the first cleavage step occurs between the enzymatic subdomains p18 and p10 and thus generating two cleavage intermediates: p43/p41 and p12. The second cleavage step of p43/p41 produces the active enzyme subunits p18, p10 and prodomain p26/p24. The two cleavage products, p43/41 and p18, can cleave downstream substrates of caspase-8. The cleavage activity of the membrane-bound p43/41 is restricted to the plasma membrane while the subunits p18 and p10 form a heterotetramer p18_2_-p10_2_, which enters into the cytosol as active caspase-8 and cleaves various target substrates^12^. In the current study, we investigated the ability of Us11 to interact with caspase-8 and modulate its activity in immune cells, particularly in monocytes, which represent the first line of defense against viruses and show appreciable apoptosis response following HSV infection. Indeed, HSV-1 infection in THP-1 cells (human acute monocytic leukemia cell line) gives rise to a two-cell population: one that gets infected and replicates the viral genome, and the second that remains resistant to HSV-1 infection. This is not surprising due to the fact that HSV-1 infects several cell types *in vitro* with different degrees of permissiveness and immune cells, including monocytic cells, macrophages, dendritic cells and T lymphocytes are less permissive to HSV-1 infection and viral replication than other cell types, such as epithelial cells. In addition, differently from permissive epithelial cells in which HSV-1 triggers necrosis as the main cell death mechanism, in the monocytes cells, viral infection induces cell death via apoptosis^13–16^. The fact that HSV-1 differentially modulates the apoptotic response, as well as the infection and survival in epithelial (permissive) and immune (semi-permissive) cells, could represent a relevant mechanism for innate immune escape and virus dissemination. Therefore, a comprehensive understanding of the interaction between the virus and the host cells is of crucial importance.

## Results

### The viral protein Us11 promotes the accumulation of a second cleavage product of caspase-8, p18, in several cell models

**Figure 1:**
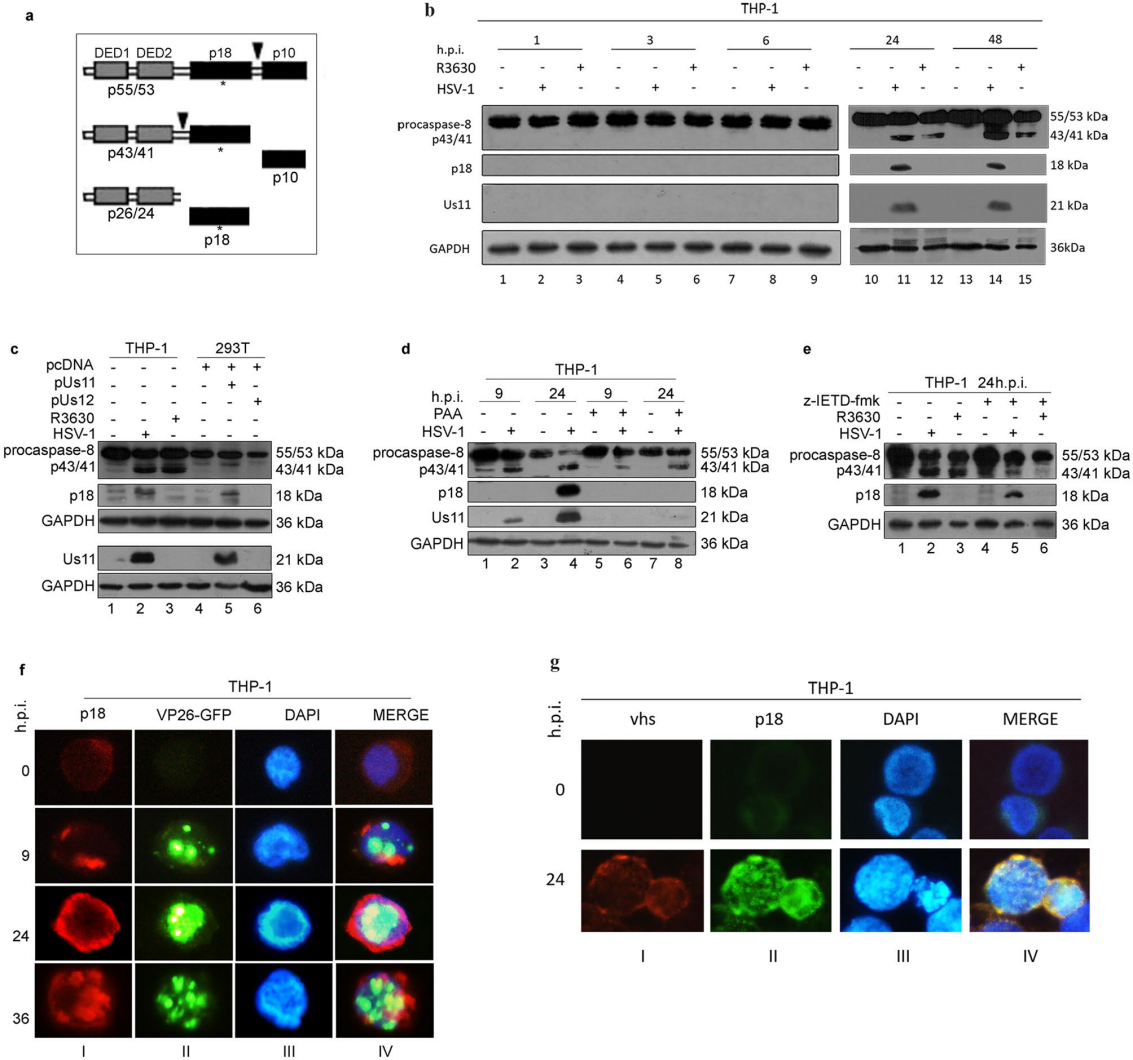
Cleavage and activation of caspase-8 during HSV-1 replication in THP-1 and effect of Us11 deletion. (**a**) Schematic representation of the “two-step” model proposed for cleavage and activation of caspase-8; (**b**) time- course analysis of caspase-8 in THP-1 cells infected or not with HSV-1 and R3630 (ΔUs11/Us12) viruses; (**c**) analysis of caspase-8 cleavages in THP-1 cells infected or not with HSV-1 or R3630 (ΔUs11/Us12) for 24 h and 293 T cells transfected with pUs11 and pUs12 plasmids and collected at 72 h post-transfection; (**d**) analyses of caspase-8 cleavage in THP-1 cells infected with HSV-1 and treated or not with PAA (300 µg/ml); (**e**) analysis of caspase-8 cleavage in THP-1 cells infected with HSV-1 or R3630 (ΔUs11/Us12) and treated or not with the caspase-8 inhibitor z-IETD-fmk (100 µM); The full-length (p55/53) and cleaved form (p43/41 and p18) of caspase-8 was detected by using a specific antibody directed to the p18 subunit (ALX-804–242-12F5). GAPDH was used as loading control; (**f**) fluorescence microscope analysis of p18-fragment in THP-1 infected with HSV-1-VP26GFP. The cells were infected or not with HSV-1-VP26GFP and stained with anti-p18 antibody in red (I). The green dots are representative of autofluorescent VP26GFP protein (II). Hoechst was used to stain the nuclei (III); the IV column represents the merged images; (**g**) co-localization of p18 with the late gene product VHS. THP-1 cells were infected or not with HSV-1 and stained with both anti-p18 (II) and anti-VHS antibodies (I). Hoechst was used to stain the nuclei (III); the IV column represent the merged images. Magnification of images × 63. The b and c were cropped from different expositions of the same gel to improve the clarity and conciseness of the presentation. Original Western blot and corresponding images of the fluorescence microscope analysis showed in f and g are presented in the Supplementary file (Fig. S1).

As discussed above, Us11 has been shown to exert a role in counteracting apoptotic signaling as well as to inhibit autophagy through its interaction with PKR in HeLa cells^8,9^*.* Based on these data, we aimed to assess its capability to regulate cellular permissiveness by recruiting caspase-8 in monocytes. A recombinant R3630 (ΔUs11/ΔUs12) virus was used to infect the human acute monocytic leukemia cell line (THP-1). THP-1 cells were infected or not with the wild-type HSV-1 and the recombinant R3630 virus to detect the caspase-8 pro-domains. The canonical “two-step model” activation of caspase-8 requires a first cleavage step between the enzymatic subdomains p18 and p10, generating the p43/p41 and p12 cleavage intermediates, and a second cleavage of the p43/p41 generating the active enzyme subunits p18, p10 and pro-domain p26/p24 (Fig. 1a). The active p43/41 and p18 cleavage products were detected by specific antibody directed to the p18-fragment. Results from a time-course infection in THP-1 cells showed a different cleavage pattern of caspase-8 in absence of Us11 protein (Fig. 1b). Particularly, the cleaved form p43/p41 was detected in both HSV-infected and R3630-infected cells compared to the uninfected cells (Fig. 1b; lanes 11-12 vs lane 10; lanes 14-15 vs lane 13) at late stage of infection (24 and 48 h post infection or p.i.). Interestingly, the fully-active p18 cleavage product was only detected in HSV-infected cells (24 and 48 h p.i.), but not in R3630 infected cells, suggesting that the lack of Us11 and Us12 viral proteins could affect the second cleavage step (Fig. 1b; lane 11 vs lane 10-12; lane 14 vs lanes 13-15). Given the fact that R3630 virus carries a deletion in both Us11 and Us12 genes, transfection experiments were included to verify the pattern of caspase-8 cleavage observed in R3630 infected cells. Transfection experiments were carried out in 293T cells, which exhibited a similar THP-1-phenotype upon infection (data not shown). The results confirmed that the overexpression of Us11 but not Us12, specifically induces p18 accumulation in transfected cells (Fig. 1c; lane 5 vs lane 6). Interestingly, the p43/41 fragment was not detected in 293T transfected cells, as observed instead in THP-1 cells, infected with HSV-1 and R3630 (Fig. 1c; lanes 5-6 vs lanes 2-3). These data were further confirmed by the inhibition of the accumulation of γ_2_ proteins, including Us11, by phosphonoacetic acid (PAA) treatment. The obtained results showed that Us11-mediated accumulation of p18 was abrogated in treated infected cells compared to non-treated infected cells. Conversely, the p43/41 fragment was detected in treated or not infected cells (Fig. 1 d; lanes 2-4 vs lanes 6-8). This evidence indicates that during infection, even in absence of Us11, the first cleavage step of caspase 8 occurs and generates the p43/41 fragment. Besides, in both infected or transfected cells, Us11 promotes an accumulation of p18, which does not require p43/41 fragment. These results were corroborated by data obtained using a caspase-8 inhibitor z-IETD-fmk, which blocks the canonical caspase-8-cleavage. As shown in Figure 1e, this alternative cleavage of caspase-8 required viral Us11 protein and was partially inhibited by z-IETD treatment (Fig. 1e lane 5 vs lane 2). Additionally, fluorescence microscope analyses were performed to verify the accumulation of p18-fragment in actively replicating cells. To this purpose, THP-1 cells were infected or not with a recombinant virus expressing the viral capsid VP26 tagged with GFP (HSV-1-VP26GFP)^19^. As shown in Figure 1f, uninfected cells showed fluorescence corresponding to the basal level of caspase-8. Unlike, a greater accumulation of p18 dots was observed consistently with the increase of autofluorescent-VP26-GFP-dots in the infected cells. This finding indicates that p18-fragment accumulates during active HSV-1 replication (Fig. 1f; columns I, II and IV). Indeed, at least 50% of infected cells GFP^**+**^ cells were positive for p18 as well GFP^**+**^/caspase8^**+**^. Uninfected cells showed a diffuse fluorescence corresponding to the basal level of inactive form of caspase-8. Moreover, to confirm these data, double staining of THP-1 cells infected with HSV-1 was performed against the active p18 fragment and the late gene product *VHS*. As shown in Fig. 1g, the co-localization of p18 and *VHS* observed in HSV-infected cells confirms that the accumulation of p18-fragment occurred in actively replicating cells (Fig. 1g; column IV). The original western blot and fluorescence microscopy images are reported in the Supplementary file 1 (Fig. S1).

### Us11 binds to p18 cleaved fragment and induces its accumulation in a cell-free system

**Figure 2:**
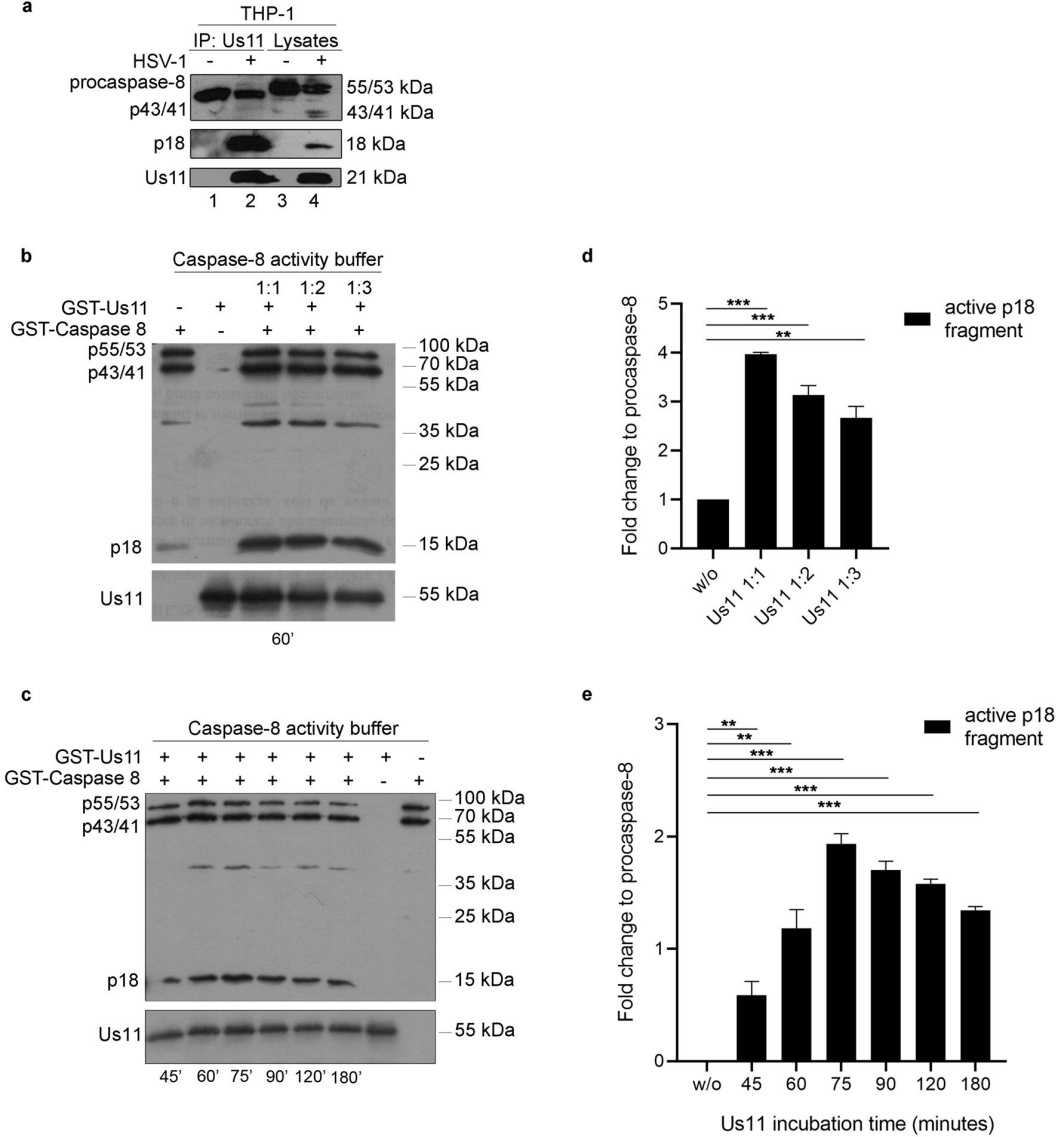
Immunoprecipitation and caspase-8 cleavage assay. (**a**) THP-1 cells were infected or not with HSV-1, lysed and then incubated overnight at 4 °C with 5 μl of Us11 antibody pre-adsorbed on protein-A Sepharose Beads. After overnight incubation with the extracts, complexate-beads were resolved by SDS-PAGE and transferred to nitrocellulose membranes. To improve the clarity and conciseness of the presentation, the figure was presented as cropping parts of the same gel first blotted with anti-caspase-8 antibody and then with anti-US11. (**b**) GST-caspase-8 recombinant protein was incubated with a serial dilution of GST-Us11 recombinant protein (1:1, 1:2, 1:3) in caspase-8 assay buffer as described in material and methods. After 1 h incubation time, the activation of caspase 8 was verified by SDS-PAGE; (**c**) GST-Caspase 8 recombinant protein was incubated with GST-Us11 recombinant protein (1:1) in caspase-8 assay buffer for a different time from 45 min up to 3 h. After the incubation time, the activation of caspase 8 was verified by SDS-PAGE; (**d**) and (**e**) The graphs represent the relative fold change of p18 band intensity over the procaspase-8 band intensity; The membranes were probed with antibodies directed to caspase-8 and Us11 proteins. The membranes were probed with a specific antibody directed to the p18 subunit and a polyclonal antibody directed to Us11. The asterisks (*, ** and ***) indicate the significance of *p*-values less than 0.05, 0.01 and 0.001, respectively.

Results obtained in Fig. 1 demonstrated that Us11 promotes a p18 cleavage of caspase-8. To verify whether the p18 cleavage was mediated by the interaction with the viral protein Us11, the physical interaction between Us11 and caspase-8 proteins was confirmed by the immunoprecipitation assay. The data clearly showed that Us11 physically interacts and precipitates the p18-fragment (Fig. 2a; lane 2). Lastly, to verify a direct connection between Us11 protein and p18 cleavage, we performed an in vitro cleavage assay using recombinant caspase-8 and Us11 proteins in a cell-free system. The western blot analysis indicated that the presence of GST-Us11 protein increases the accumulation of p18-fragment from GST-caspase-8 in a cell-free system (Fig. 2 panels b and c). In particular, the p18-fragment accumulates in a dose- and time dependent-manner in GST-Us11-incubated samples compared to GST-caspase-8 alone as indicated in Fig. 2 panels d and e. Therefore, Us11 is able to interact with caspase-8 and promote its cleavage in both an in vitro and in a cell-free system. Moreover, it is believed that upon their interaction, Us11 and the active form p18 remain physically associated as suggested in the immunoprecipitation analysis. The original western blot figures are reported in Supplementary file 2 (Fig. S2).

### Us11-dependent p18 accumulation does not trigger apoptosis

**Figure 3:**
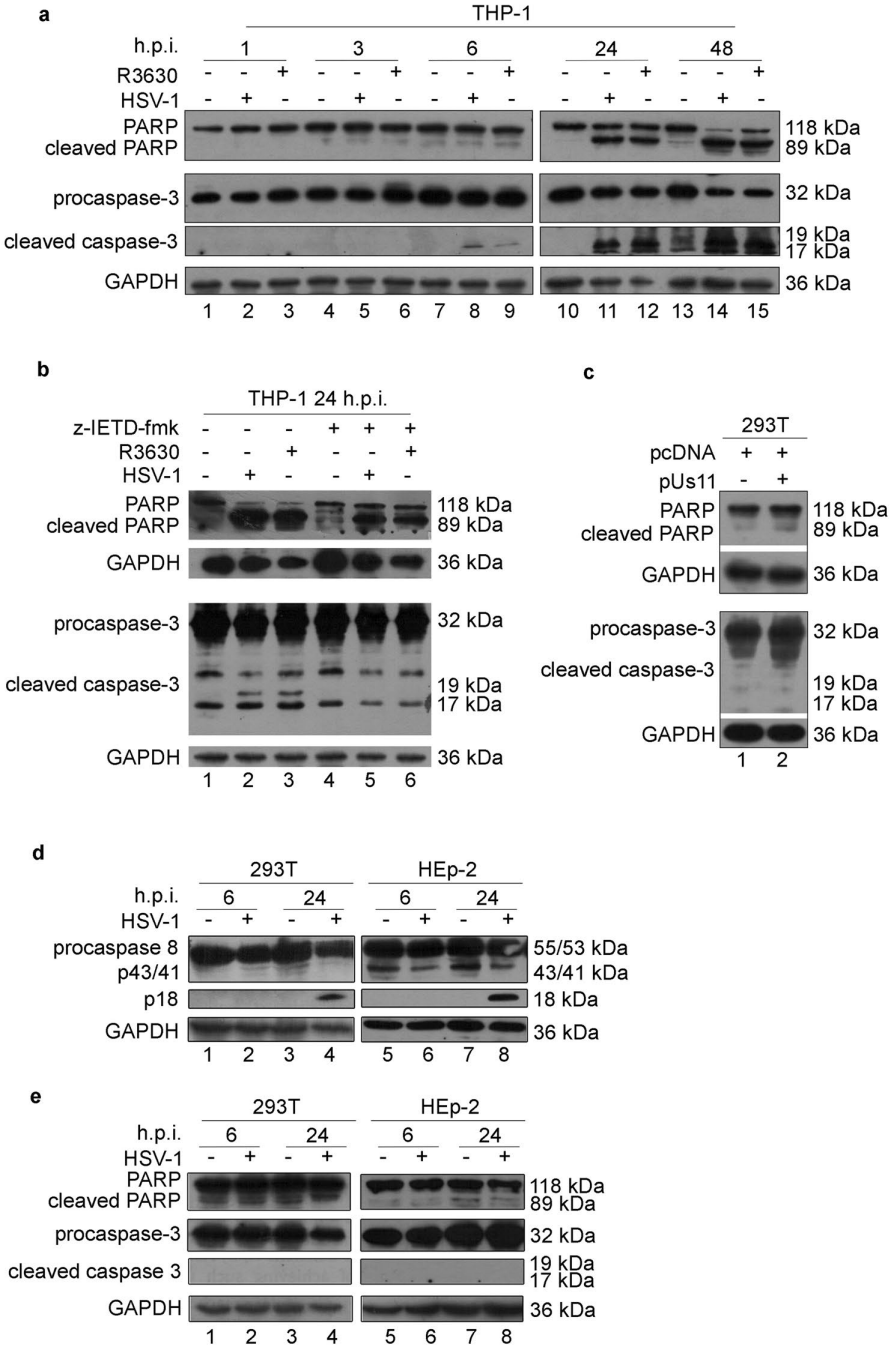
Analysis of apoptotic markers. Western blot analysis of PARP and caspase-3 from: (**a**) THP-1 cells infected with HSV-1 or R3630 (ΔUs11/Us12) and collected at 1,3,6,24 and 48 h.p.i; The grouping blots are cropped from two different simultaneously exposed gels. (**b**) THP-1 cells infected with HSV-1 or R3630 (ΔUs11/Us12) and treated or not with the caspase-8 inhibitor z-IETD-fmk (100 µM); (**c**) THP-1 cells transfected with pUs11 and collected 72 h post transfection. The membranes were probed with an antibody directed to caspase-3 and PARP. GAPDH was used as a loading control; The grouping blots are cropped from two different gels as reported in supplementary file 3 (Fig. S3) (**d**) and (**e**) analysis of caspase-8 and apoptosis marker in non-immune cells (293 T and HEp-2) infected or not with HSV-1. To improve the clarity and conciseness of the presentation, the figures were presented as cropping parts of the same gel (Fig. S3). The membranes were probed with antibodies directed to caspase-8, caspase-3 and PARP. GAPDH was used as a loading control.

To verify whether the alternative cleavage of caspase-8 observed in HSV-infected and Us11-transfected cells differentially modulates the cell death signaling, we investigated the apoptosis pathway through the detection of cleaved caspase-3 (17-19kDa) and cleaved poly(ADP-ribose) polymerase-1 (PARP)(118-89 kDa). A time-course analysis in THP-1 cells indicates that in both wild-type HSV-1 and R3630 infected cells the cleavage of caspase-3 and PARP occurred at later time point post-infection (24 and 48 h.p.i) confirming the activation of the apoptotic pathway (Fig. 3a; lanes 11-12 and 14-15). However, these results suggested that the different cleavage pattern of caspase-8 observed between the HSV-1 and R3630 infected cells does not result in a different cleavage of caspase-3 and PARP. Indeed, by preventing the canonical caspase-8 cleavage with the z-IETD-fmk inhibitor, we found that cleaved caspase 3 (19-17 kDa) and PARP (89 kDa) accumulation were inhibited by z-IETD-fmk treatment in both HSV-1 and R3630 infected cells (Fig. 3b; lanes 5-6 vs lanes 2-3). This evidence clearly confirmed that the canonical cleavage of procaspase-8, induced during HSV-1 and R3630 infection, results in apoptosis induction as confirmed by p43/41-dependent PARP and caspase-3 cleavage. Conversely, the accumulation of p18, dependent on Us11 expression, was not related to apoptosis activation. Indeed, overexpression of Us11 in non-infected cells does not result in caspase-3 and PARP cleavage (Fig. 3c; lane 2). Differently from monocytic cells, in which HSV-1 infects with different degrees of permissiveness and appears to induce their cell death via apoptosis^13–16^, in epithelial cells, HSV-1 prevents apoptosis by causing cell death with predominant features of necrosis. Thus, it was subsequently verified whether the p18-cleavage triggers apoptosis in permissive epithelial cells upon HSV-1 infection. As shown in Fig. 3d and e, p18 accumulation was observed in both 293T and HEp-2 infected cells at 24 h.p.i (Fig. 3d; lane 4 and 8), while neither p43/41 nor cleaved PARP and cleaved caspase-3 were detected (Fig. 3e). Overall, the results obtained from these experiments demonstrated that Us11-dependent accumulation of p18 does not enhance apoptotic response during HSV-1 infection either in semi-permissive as well as in permissive cell lines. The original western blot data are reported in the Supplementary file 3 (Fig. S3).

### Cleavage of Atg3 protein by activated-caspase-8 during HSV-1 replication results in LC3II lipidation

**Figure 4:**
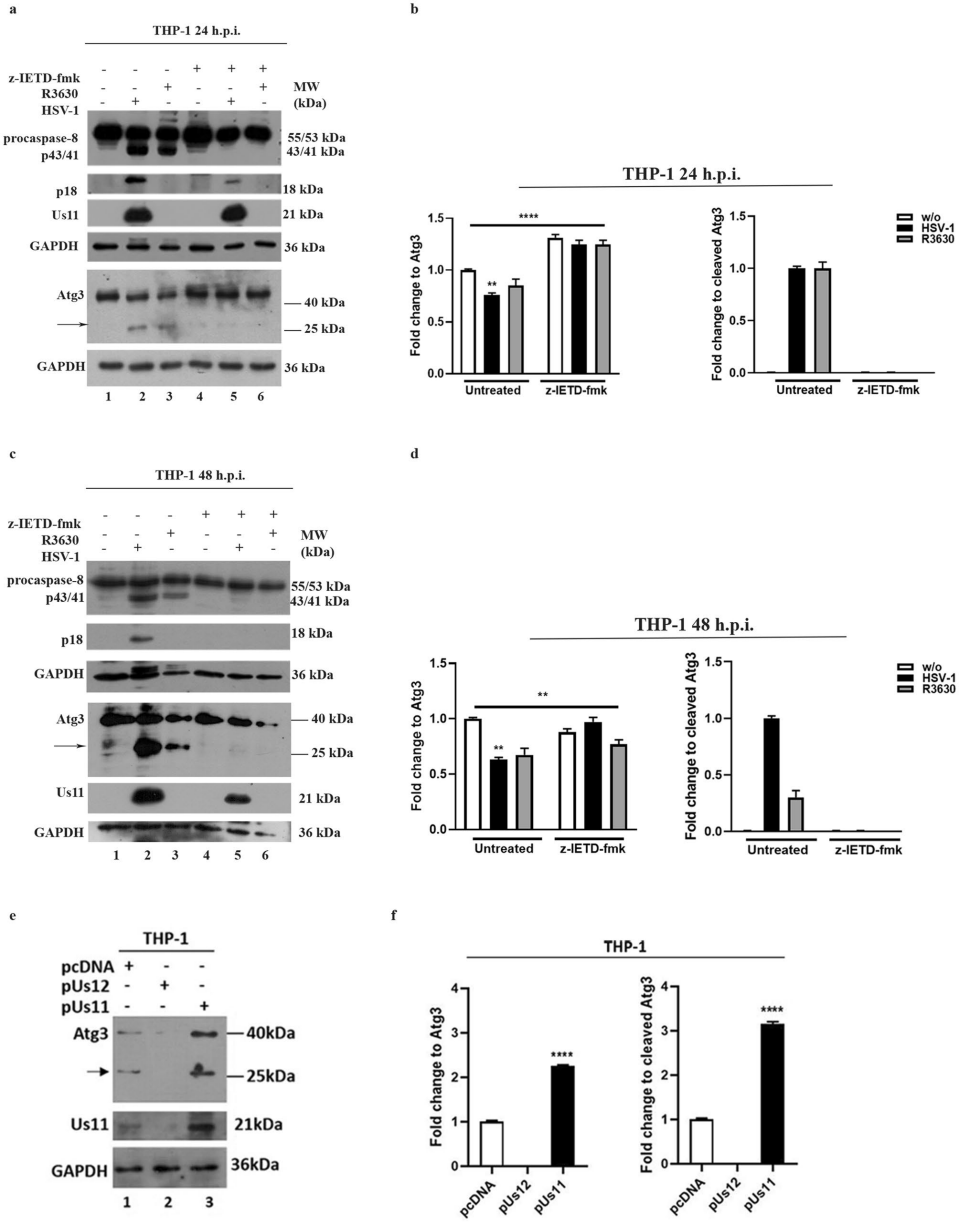
Cleavage of Atg3 protein in infected and transfected THP-1 cells. (**a**–**c**) THP-1 cells were infected with HSV-1 or R3630 (ΔUs11/Us12), treated or not with caspase-8 inhibitor z-IETD-fmk (100 µM) and collected 24 h and 48 h p.i. Atg3 degradation was analyzed by immunoblotting. GAPDH was used as a loading control. (**b**–**d**) Band intensity of Atg3 and fragments was determined with the T.I.N.A. program, expressed as fold change over the appropriate housekeeping genes and graphically represented with GraphPad Prism 6 software. Statistical significance was tested using one-way ANOVA (**p < 0.01). (**e**) THP-1 cells were transfected with pUs11 and pUs12 plasmids and collected 48 h post-transfection. (**f**) Band intensity of Atg3, Atg3 fragment was determined with the T.I.N.A. program, expressed as fold change over the appropriate housekeeping genes and graphically represented with GraphPad Prism 6 software. Statistical significance was tested using one-way ANOVA (****p < 0.001) Original Western blots are reported in Supplementary file 4 (Fig. S4).

In addition to its conventional role, it has been demonstrated that caspase-8 can cleave Atg3, an E2-ubiquitin-like conjugating enzyme involved in autophagosome formation favoring cell survival and that this event seems to be the main mechanism of autophagy inhibition by death receptor activation^17^. Indeed, the Authors demonstrated that tumor necrosis factor-induced cell death was accompanied by Atg3 cleavage and that a caspase-8-specific inhibitor (zIETD) blocked this event. In this contest, to understand the biological role of Us11-dependent p18 accumulation we analyzed Atg3 protein as a representative substrate of activated caspase-8 protein during HSV-1 replication. We found that HSV-1, in THP-1 infected cells, specifically cleaves Atg3 protein as shown by the reduction in the full-length protein compared to uninfected cells and by detecting cleavage fragments (Fig. 4a lanes 2-3 and Fig. 4b). Otherwise, the addition of the caspase-8 inhibitor z-IETD-fmk resulted in the block of Atg3 cleavage suggesting a direct connection between the activation of caspase-8 mediated by HSV-1 and Atg3 degradation (Fig. 4a lanes 5-6 and Fig. 4b). Interestingly, we found that Atg3 cleavage fragment strongly accumulates at 48h post-HSV-1 infection but not by R3630 suggesting that the cleavage is dependent on Us11-mediated p18 accumulation (Fig. 4c lanes 2 vs 3 and Fig. 4d). To confirm that the Us11-dependent accumulation of p18 correlates with Atg3 cleavage, we performed a transient transfection on THP-1 with the plasmid encoding for Us11 and Us12, separately. We found that the transfection with Us11, unlike to Us12, induces the accumulation of Atg3 protein and its cleavage compared to the transfection control (Fig.4e lanes 3 vs 1 and Fig. 4f). The expression of Us11 protein is also shown (Fig. 4e lanes 3).

**Figure 5:**
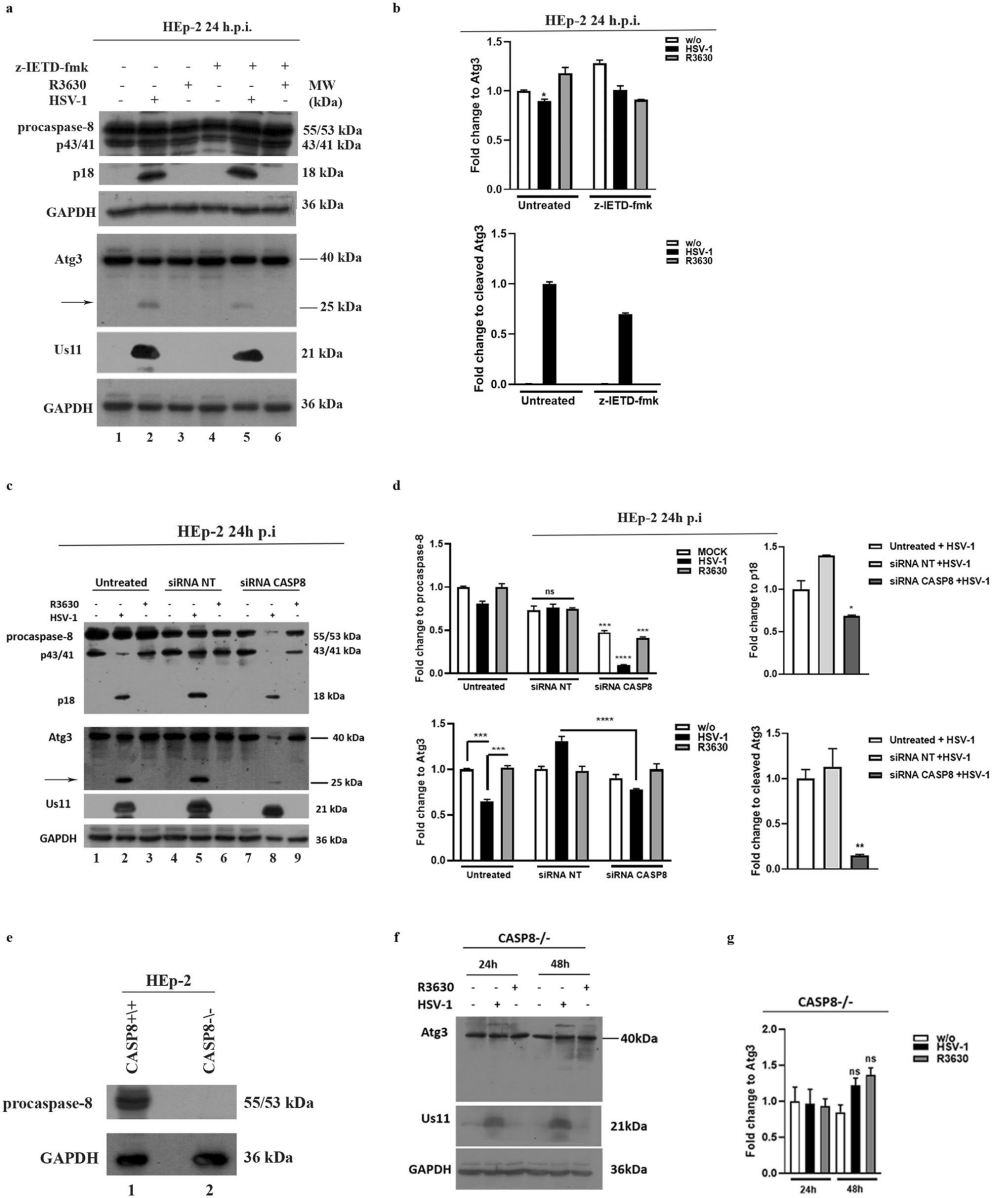
Cleavage of Atg3 protein in HEp-2 infected cells (**a**) HEp-2 cells were infected with HSV-1 or R3630 (ΔUs11/Us12), treated or not with caspase-8 inhibitor z-IETD-fmk (100 µM) and collected 24 h p.i. Atg3 degradation was analyzed by immunoblotting. GAPDH was used as a loading control. (**c**) Knockdown of Caspase-8 was performed using a pool of siRNAs. HEp-2 (2.5 X 10 5 cell/well) were seeded into 6 well plates for 24 h. Then, 300 nM of each siRNAs targeting a different region of caspase-8 (siRNA CASP8) or negative control siRNA (siRNA NT) were transfected on HEp-2 cells. Untransfected infected cells were set as control. Then, 48 h post-transfection the cells were infected for a further 24 h with HSV-1, R3630 (ΔUs11/Us12) or not and collected for immunoblotting. Arrows indicate the cleavage fragments. Original Western blots are reported in Supplementary file 5 (Fig. S5). (**e**) Immunoblot analyses of lysates of HEp2 Caspase-8 knocked-out cells (CASP8^-\-^) and wild type (CASP8^+\+^) for accumulation of knocked-out genes. (f) CASP8^-/-^ cells were infected with HSV1 or R3630 (ΔUs11/Us12), and collected 24 h and 48 h p.i. Atg3 cleavage was analyzed by immunoblotting. GAPDH was used as a loading control. (**b**,**d**,**g**) Band intensity of Atg3, Atg3 fragment, procaspase-8 and p18-fragment was determined with the T.I.N.A. program, expressed as fold change over the appropriate housekeeping genes and graphically represented with GraphPad Prism 6 software. Statistical significance was tested using one-way ANOVA (****p < 0.001).

**Figure 6:**
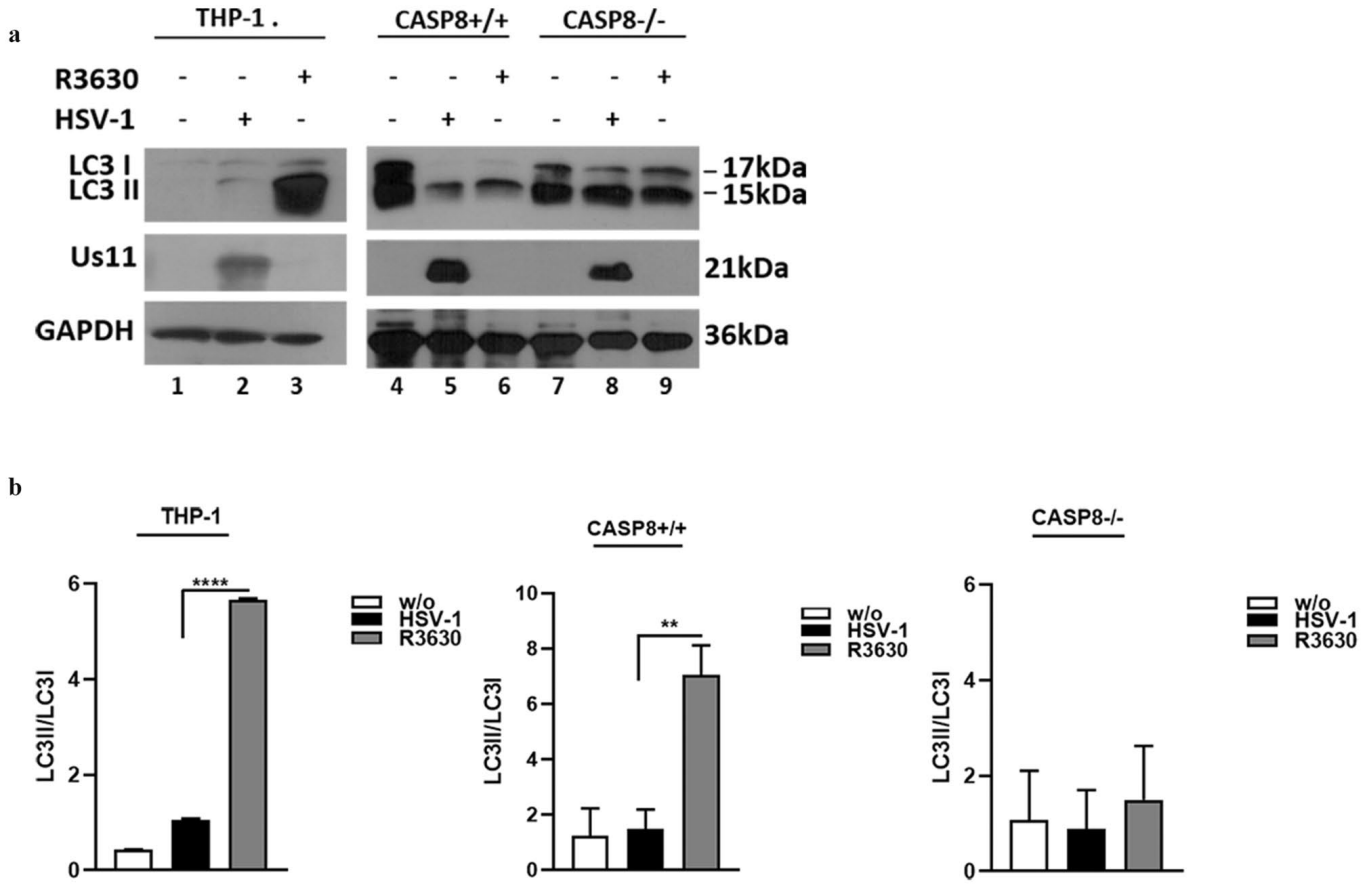
LC3 lipidation in HSV-1 infected cells. (**a**) THP-1 cells were infected at 50 MOI with HSV-1 and R3630 and collected 48 h p.i. CASP8^+/+^ and CASP8^-/-^ cells were infected at 10 MOI with HSV-1 and R3630 and collected 24 h p.i. Cells were then lysed and the LC3 expression was detected by immunoblotting. GAPDH was used as a loading control. (**b**) The LC3-II/LC3-I ratios of HSV-1 and R3630 infected samples were normalized to the LC3-II/LC3-I ratio of control-uninfected cells and graphically represented with GraphPad Prism 6 software. Statistical significance was tested using one-way ANOVA (**p < 0.01, ****p < 0.001).

Similarly, we detected the Atg3 cleavage fragment in HEp-2 cells following HSV-1 infection and depending on Us11-mediated p18 accumulation (Fig. 5a lanes 2 vs 3 and Fig. 5b) as shown by the detection of no cleavage fragment by R3630 infection. However, the HEp-2 cells treated with the zIETD inhibitor did not show a clear reduction in caspase 8. Besides, to verify the involvement of caspase-8 in ATG3 cleavage, we used a double approach. Thus, we used a pool of chemically synthesized siRNAs to knockdown caspase-8 (Fig. 5c,d) and enrolled caspase-8 deficient HEp-2 cells (CASP8^-/-^) which were validated by immunoblot (Fig. 5e). We observed that HEp-2 infected cells, where caspase-8 was silenced, did not show the Atg3 cleavage fragment compared to the infected control cells (Fig. 5c lane 8 vs 2). Similar results were obtained in CASP8 ^-/-^ cells (Fig. 5 panels f and g). These findings collectively confirm that the activation of caspase 8 by p18-fragment accumulation allows the downstream Atg3 cleavage. The original western blot figures are reported in the Supplementary file 4 and 5 (Fig. S4 and S5). Lastly, because Atg3 degradation correlates with the attenuation of autophagy, we verified the Microtubule-associated protein light chain 3 (LC3) lipidation upon HSV-1 infection and detected the LC3 conversion (LC3-I to LC3-II) by immunoblot analysis considering that the amount of LC3-II is correlated with the number of autophagosomes^18^. We detected the lipidation of LC3 on THP-1 cells and CASP8^+/+^ by reporting an increased LC3-II/LC3-I ratio following R3630 infection in THP-1 and CASP8^+/+^ cells when compared to the HSV-1 infected cells or to the CASP8^-/-^ cells (Fig. 6a lanes 3 vs 2 and 6 vs 5 and Fig 6b). These data validate the hypothesis that the Atg3 cleavage, downstream of Us11-mediated p-18 accumulation, could be associated with a block of autophagy. The original western blot result is reported in supplementary file 6 (Fig. S6).

### Role of Caspase-8 protein in HSV-1 replication cycle

**Figure 7:**
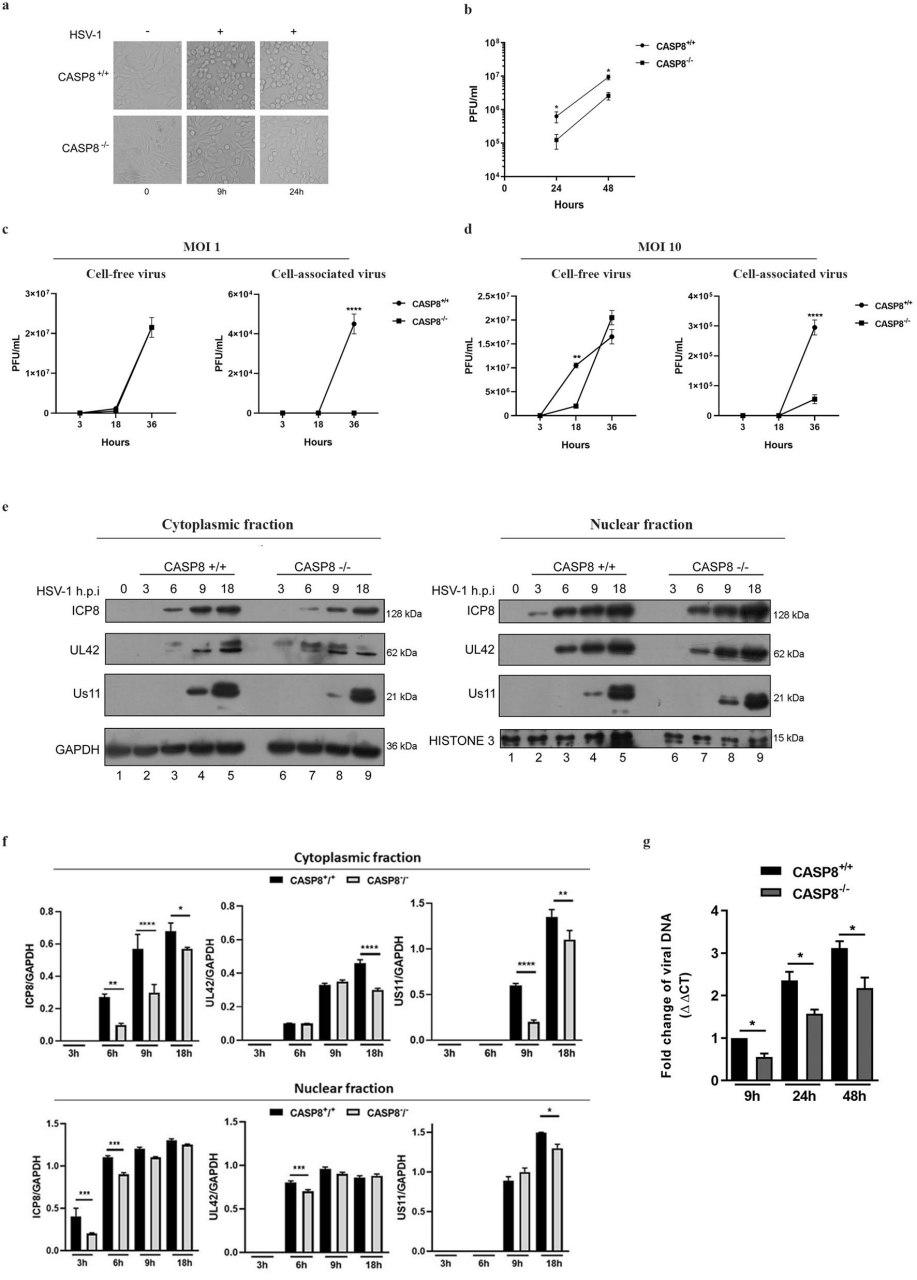
Comparison of HSV-1 replication efficiency in CASP8^+/+^ and CASP8^-/-^ cells. Caspase-8 deficient (CASP8^-/-^) and the wild type (CASP8^+/+^) cell lines were infected with HSV-1 and collected at several times p.i. (**a**) the cytopathic effect was observed under inverted light microscopy (magnification × 10) at 9 h and 24 h.p.i; (**b**) samples were collected at 24 and 48 h.p.i and the virus yield was evaluated by total viral particle titration; (**c**,**d**) titration of cell-associated virus and cell-free virus particles on VERO cells; (**e**) equal amount of cytoplasmic and nuclear proteins were resolved by SDS-PAGE and the membranes were probed with antibodies directed against the viral protein ICP8 (β), UL42 (β) and Us11 (γ2). GAPDH and Histone 3 were used as a loading control (**f**); quantitative densitometry analysis of immunoblot band intensities was performed with the TINA software (version 2.10, Raytest, Straubenhardt, Germany) and it is expressed as the fold change over the appropriate housekeeping gene; (**g**) relative quantification of viral DNA was performed by using specific HSV-1 TaqMan probe and analyzed by the comparative Ct method (ΔΔCt). Original Western blot and microscopy images are reported in supplementary file S5 (Fig. S5). The asterisks (*, **, *** and ****) indicate the significance of *p*-values less than 0.05, 0.01, 0.001 and 0.0001, respectively.

To investigate the role of Caspase-8 in HSV-1 replication, we firstly compare the cytopathic effect on CASP8^+/+^ and CASP8^-/-^ cells. The HSV-1 infection exhibits a different phenotype between the two cell lines as shown in Fig. 7a. At 9h and 24h post-infection (multiplicity of infection; MOI 10), the distinctive cytopathic effect (round cells/detached cells), due to HSV-1 replication, was detected in the wild type cells expressing caspase-8 (CASP8^+/+^), while the CASP8^-/-^ cells showed a less evident cytopathic effect. Afterwards, the viral title was quantified by plaque assay. In Fig. 7b, total viral particle titration confirms the data reported in Fig. 7a. Next, we investigated whether there was a difference in the release of mature viral particles in the two cell lines using two different MOI. Virus titer was determined from cell-free and cell-associated supernatants by plaque assay on VERO cells. The results indicate that at MOI 1 and 10, the cell-free virus titers were greater in CASP8^+/+^ rather than CASP8^-/-^ (Fig. 7 panels c and d). Interestingly, at MOI 10 an increase of the cell-associated virus titer was observed in CASP8^+/+^ rather than CASP8^-/-^ only at 18h p.i. While a boost of the cell-free virus was observed in CASP8^+/+^ rather than CASP8^-/-^ at 36h p.i. Next, viral proteins expression was checked in CASP8^+/+^ and CASP8^-/-^ cells following HSV-1 infection at 10 PFU/cells. The cells were collected at 3, 6, 9 and 18h p.i. to evaluate the expression of the viral proteins ICP8 (β), UL42 (β) and Us11 (γ2) which are representative viral products of HSV-1 proteins cascade. Analysis of cytoplasmic and nuclear fractions revealed a different accumulation of the viral proteins in CASP8^-/-^ cells when compared to CASP8^+/+^ (Fig. 7 panels e and f). Interestingly, for ICP8 viral protein, there was a delay of protein accumulation in both fractions in all-time considered in CASP8^-/-^ in comparison to CASP8^+/+^ (Fig. 7e, lanes 7-9 vs lanes 3-5). UL42 viral protein was mainly localized in the nuclear fraction in both cell lines. However, the data indicate a less accumulation of UL42 in CASP8^-/-^ related to CASP8^+/+^ cells at 6h p.i. Being UL42 involved in the replication of HSV-1, this result can justify the decrease in viral particles production. Concerning Us11 accumulation, a difference was detected between the CASP8^-/-^ compared to CASP8^+/+^ cells. In particular, at 9h p.i. Us11 protein localizes mainly in the nuclear fraction of CASP8^-/-^ cells compared to CASP8^+/+^. Furthermore, the amount of viral DNA suggested that in CASP8^+/+^ cells the accumulation of viral DNA was significantly higger compared to CASP8^-/-^ cells (Fig. 7g). Given the fact that the  highest differences between CASP8^+/+^ and CASP8^-/-^ cells lines were observed in the released virus particles, these results indicate that activation of caspase-8 protein could be crucial during the late phase of HSV-1 replication, leading to hypothesize a caspase-8 contribution during the viral maturation. The original western blot data and microscopy images are reported in the Supplementary file 7 (Fig. S7).

## Discussion

Immune cells represent the first line of defense against viral pathogens. In this scenario, the apoptosis pathway represents an innate immune response activated in order to restrict viral replication. In this regard, HSV-1 has evolved several immune escape strategies which involve the production of virulence factors^19^. The immune cells such as lymphocytes, monocytes/macrophages and dendritic cells, sustain a low-productive infection characterized by induction of apoptosis as cytopathic effect. However, the molecular mechanisms that control the restriction of HSV-1 replication in immune cells are not completely understood. Indeed, it has been reported that HSV-1 induces the early activation of autophagy in human monocytic THP-1 cells, most likely promoting viral internalization. Conversely, autophagy is belatedly inhibited or stabilized to support efficient viral replication^20^. HSV-1 is well known to differentially modulate apoptosis through the expression of several viral proteins which interfere with host antiviral responses. The viral encoded Us11 late protein has been shown to protect HeLa cells from heat- and staurosporine-induced apoptosis as well as to counteract the autophagy response^8,9^. Therefore, in this work, the interaction between Us11 and caspase-8 was explored in a monocytoid cell system, THP-1, which are sensitive to apoptosis signaling^16^. Caspases-8 is the initiator caspase of extrinsic or death receptor-mediated apoptosis in mammals. It is also implicated in other cell death processes like necroptosis and autophagy and acts as a switch point between autophagy and apoptosis, exerting an important role in cell-fate determination and immune response. The results obtained from this study demonstrate that Us11 is able to directly induce a non-canonical cleavage of caspase-8, bypassing the first cleavage step and releasing p18-fragment only. An alternative cleavage model has been also described by Hoffmann and colleagues in B-lymphoblastoid cell lines SKW6.4, Raji, and BJAB and the T-cell lines CEM and Jurkat 16, upon CD95 stimulation^21^. The authors described a first cleavage between the prodomain and the large enzymatic subunit p18, which generates the p26/p24 and p30 products. Afterwards, additional cleavage of p30 by active caspases (caspase-8 and -9 but not by caspase-3) allowed to p18 and p10 release. Furthermore, p30 can sensitize cells toward death receptor-induced apoptosis. Here, we reported that the cleavage of caspase-8 mediated by the Us11 protein was observed not only in monocytes but also in epithelial cells and was not related to a downstream modulation of the apoptosis pathways. These findings suggested that different activation pathways could trigger caspase-8 enzymatic activity and/or may promote the switch between caspase-8 apoptotic and non-apoptotic functions. To date, the initial concept of an initiator caspase in extrinsic cell death signaling was replaced and expanded with the new regulatory role in cell survival and in several inflammatory processes^22^. Thus, we examined Atg3, autophagy-related protein as a target of caspase-8 during HSV-1 replication. We observed that HSV-1 triggers the Atg3 degradation with a caspase-8-dependent mechanism, considering that the caspase-8-specific inhibitor blocked Atg3 cleavage. Post-translational modifications of Atg3 were previously reported by Norman and collaborators that described the in vitro cleavage of Atg3 by caspase-8, caspase 3 and caspase-6^23^. We demonstrated that HSV-1 recruits Us11 protein to stimulate the activation of caspase-8 by p18-fragmentation that culminated downstream in Atg3-cleavage. Besides, the pharmacological inhibition of caspase-8 and the siRNA gene silencing established a direct connection between the Atg3 degradation and p18-activation of caspase-8. Unlikely, the lack of US11 moderately restores the Atg3 full length inhibiting its cleavage (Figs. 4 and 5). Our previous studies reported that the early activation of autophagy-mediated by HSV-1 in semi-permissive THP-1 cells and human monocytes had a proviral role most likely promoting viral internalization. However, its inhibition was required during the final phase of the viral replication cycle, supporting the idea that autophagy functions as a restriction mechanism against viral infections^20^. Indeed, it is known that HSV-1 counteracts the autophagic response in different cell lines^24,25^ through the direct interaction between γ34.5 and Beclin1^26^. Lussignol and colleagues demonstrated that both viral proteins, Us11 and γ 34.5, negatively regulate the autophagy process during the viral life cycle by acting on antiviral signaling molecule PKR which is upstream of Beclin 1 in host antiviral defense^9^. Based on this data and on the caspase regulated-intracellular trafficking role, we hypothesized that the Atg3 degradation mediated by caspase 8 during the late phase of HSV-1 cycle could be a further potential mechanism to limit autophagy and support a better viral replication. For this reason, we employed the Caspase-8 knock-out cells to evaluate the viral replication performance. Our data reported that the lack of caspase 8 is related to less infectivity (Fig. 7). This sustains the hypothesis of the non-apoptotic function of caspase 8. Indeed, numerous studies in the literature indicated that caspase-8 exerts several non-apoptotic functions, including promotion of cell adhesion, embryonic development, monocyte differentiation, T and B cell proliferation, activation of NF-kB and tumorigenesis^27,28^. Caspase-8 expression is retained in many tumors suggesting that its apoptotic activity may switch off and its function rewired to sustain tumor growth^29^. Consistently, it has been shown that selective impairment of caspase-8 expression in T-cells leads to immunodeficiency in mice and humans as well^30,31^. Several studies have demonstrated that caspase 3 and 8 activities in monocytes modulate the delicate balance between apoptosis and myeloid differentiation following the treatment with the macrophage colony-stimulating factor (M-CSF)^32,33^. The HSV-1-mediated caspase-8 activation does not lead to apoptosis but by cleaving Atg3, could be involved in the differentiation process that requires cytoskeleton rearrangements, differential transcriptional regulation as well as changes in cell adhesion. It is known that the human cytomegalovirus (HCMV) tightly modulates the caspase 3 and to a lesser extent the caspase-8 activity to promote myeloid differentiation, a key process in the viral dissemination and persistence strategy^34^.

Therefore, the results obtained in the present study demonstrated for the first time that the viral protein Us11 interacts and modulates the cleavage of caspase-8 through a “non-canonical” mechanism not related to apoptosis response. Of relevance to this effect, we found that HSV-1 induces the late fragmentation of Atg3 in Us11 dependent manner. The Atg3- cleavage might serve as a booster escape from the autohphagic-mechanism necessary to ensure greater viral fitness. Lastly, we show an unexpected role of caspase 8 in HSV-1 infection, as a crucial regulator of extrinsic cell death, which raises new and intriguing questions for future investigation.

## Methods

### Cell lines

VERO (African green monkey kidney), 293T (human embryonic kidney), HEp-2 (human HeLa contaminant carcinoma) and THP-1 (human acute monocytic leukemia) cell lines were all originally obtained from ATCC (https://www.atcc.org/). Caspase-8 deficient HEp-2 cells (CASP8^-/-^) were gently provided by Professor Zhou G. (Shenzhen International Institute for Biomedical Research, Shenzhen, Guangdong, China). VERO cells were cultured in Dulbecco’s Modified Eagle’s Medium (DMEM) supplemented with 6% fetal bovine serum (FBS, Euroclone). 293T cells were cultured in DMEM supplemented with 10% FBS. HEp-2 and CASP8^-/-^ cells were cultured in Roswell Park Memorial Institute (RPMI)-1640 medium supplemented with 10% FBS. THP-1 cells were cultured in RPMI-1640 medium supplemented with 10% FBS (Euroclone), 1mM Sodium Pyruvate (Sigma-Aldrich), 10 mM Hepes buffer (Sigma-Aldrich). All culture media were supplemented with mixture of 100 I.U./ml penicillin and 100 µg/ml streptomycin (Lonza, Belgium). All cell lines were incubated at 37° C whit 5% CO_2_.

### Viruses

The wild type herpes simplex virus type 1 (HSV-1) and the recombinant R3630 (ΔUs11/Us12) were kindly provided by Professor Bernard Roizman (University of Chicago). HSV-1 (F) is the prototype HSV-1 strain F, whereas the recombinant R3630 virus is lacking the genes Us11 and Us12. HSV-1-VP26GFP virus, expressing a GFP tagged capsid protein VP26 was described previously^19^. Viral stocks were propagated and then titrated in VERO cells. For experimental infection HSV-1, R3630 and HSV-1-VP26GFP diluted in medium or medium alone (mock-infected) were adsorbed onto cells for 1 h at 37 °C in 5% CO_2_ with gentle shaking, at different multiplicity of infection (MOI). The inoculum was then removed and replaced with fresh medium, cells were incubated at 37°C in 5% CO_2_ and collected at the indicated times post infection (p.i.) to perform experiments. The MOI used for experimental infection was MOI 10 for HEp-2 and 293T cells and MOI 50 for THP-1 cells.

### Standard Plaque Assay on VERO cells.

The plaque assay was performed on VERO cells. The supernatants (cell-free) and cell pellets (cell-associated) infected samples were frozen and thawed three times and diluted. Hundred µl of each dilution of the suspension was used to infect the confluent monolayers. The multiwell plates were incubated for 1h at 37°C. Then, viral inoculum was removed and 1ml of culture medium containing 0.8% methylcellulose was added. After 72h the plaques were visualized and counted at the microscope after staining with a crystal violet solution.

### Protein extraction and immunoblot analysis

Cell pellets were collected at the indicated time after infection or transfection, washed in 1X phosphate-buffered saline (PBS) and lysed with cell lysis buffer (Cell Signaling Technology). To detect LC3 protein, the following lysis buffer was used: 65 mM Tris HCl pH 6.8, 4% SDS, 1.5% β-mercaptoethanol. Gels containing different percentages of SDS-polyacrylamide were used: 15% to resolve LC3 forms I and II, 12.5% for caspase-8 and 10% for Atg3 and viral proteins. An equal amount of protein extracts was subjected to Sodium dodecyl sulfate-polyacrylamide gel electrophoresis (SDS-PAGE) in polyacrylamide gels, transferred to nitrocellulose membranes (Bio-Rad Life Science Research, Hercules, CA), blocked and reacted with primary antibody and appropriate secondary antibody, followed by chemiluminescent detection. Quantitative densitometry analysis of immunoblot band intensities was performed by using the TINA software (version 2.10, Raytest, Straubenhardt, Germany).

### Antibodies and reagents

Caspase-8 (human) monoclonal antibody (12F5; ALX-804-242) directed against the p18 subunit was purchased from Enzo Life Sciences. Monoclonal anti-US11 and anti-ICP8 were provided by professor Bernad Roizman. Anti-GAPDH (sc-32233), anti-HSV-1 UL42 (sc-53333) and goat anti-mouse IgG, F(ab')2-PE (sc-3798) and anti-Atg3 (sc-393660) were purchased from Santa Cruz Biotechnology. Anti-caspase 3 (#9662), anti-PARP (#9542), GAPDH (Rabbit mAb #2118) and anti-LC3B(D11) (Rabbit mAb #3868) were provided from Cell Signaling Technology. Secondary HRP-conjugated anti-mouse IgGVeriBlot for IP were from Abcam. Secondary HRP-conjugated goat anti-mouse IgG and goat anti-rabbit IgG were purchased from Millipore. The z-IETD-FMK caspase-8 inhibitor (ab141382) was purchased from Abcam.

### Immunofluorescence assays

For immunofluorescence analysis cells were layered on polylysinated slides, fixed in 4% paraformaldehyde (PFA 4%) in PBS 1X for 15 min and permeabilized with 0.1% Triton X-100 in PBS 1X. Cells were washed three times with PBS 1X and incubated with the primary antibody for 1 h at 37 °C, followed by incubation with the phycoerythrin (PE)-conjugated anti-rabbit antibody for 1h at 37°C. Cell nuclei were stained with Hoechst 2,5 μg/ml. Samples were analyzed on a fluorescence microscope.

### Immunoprecipitation

THP-1 cells were infected or mock-infected with HSV-1 at MOI 50, collected at 18h p.i. and lysed with cold lysis buffer (20 mM Tris-HCl pH 8, 1 mM EDTA, 200 mM NaCl, 1% Nonidet P-40, 2 mM DTT, 0.1 mM Na3VO4, 10 mM NaF, 0.1 μg/ml Protease Inhibitors). The supernatants were collected and precleared with 50% of protein-A slurry for 18 h. Immunoprecipitation was performed with 5 μl of the anti-Us11 monoclonal antibody pre-adsorbed on protein A-Sepharose beads (Amersham Pharmacia Biotech AB) for 2 h at 4 °C. After overnight incubation, complexate-beads were resolved by SDS–PAGE and transferred to nitrocellulose membranes (Biorad). Immunoblotting was performed by using anti-Caspase 8 antibody (12F5 Enzo) and secondary antibodies specific for IP (Abcam).

### Construction of recombinant Baculoviruses

Full length caspase-8 isoform a was cloned into the pAcGHLT-A baculovirus transfer vector (PharMingen) derived from pAcG1 vector and containing a 6xHis tag and a glutathione S-transferase (GST) tag upstream of the MCS (multiple cloning site). An NdeI/NotI fragment containing the coding sequence of the full-length caspase-8 (NCBI GenBank: AH007578.2) was amplified by PCR from a cDNA template from THP-1 cells by using the following primers: Fw-NdeI-5’-ggcatatgcatggacttcagcagaaatctttatgatattg-3’, Rev-Casp8-NotI-5’-ttgcggccgctcaatcagaagggaacagaagtttttttc-3’. The recombinant plasmid was generated by inserting the NdeI/NotI fragment containing the Caspase-8 coding sequence into the NdeI/NotI -digested plasmid pAcGHLT-A. The Caspase-8-pAcGHLT-A plasmid sequence was analyzed after cloning. The recombinant GST-Caspase-8 baculovirus was generated by cotransfection of Sf9 insect cells with the Caspase-8-pAcGHLT-A transfer plasmids along with baculoGold DNA (PharMingen), according to the manufacturer's instructions and with the aid of the Mirus TransIT-2020 Transfection Reagent. The Us11 coding sequence from Us11-pRB5850^35^ was digested with *EcoRI*/*BglII* restriction enzyme and subcloned into pAcGHLT-A baculovirus transfer vector. The recombinant GST-Us11 baculovirus was generated by cotransfection of Sf9 insect cells with the Us11-pAcGHLT-A transfer plasmids along with baculoGold DNA (PharMingen), according to the manufacturer's instructions. All the recombinant baculoviruses were amplified in Sf9 cells and the expression of the recombinant proteins was verified by western blot analysis.

### Purification of GST-Caspase 8 proteins

The recombinant GST-Caspase 8 protein was produced by infecting Sf9 cell with the recombinant baculovirus and incubating the cells for 3 days at 27°C. The cells were then collected through centrifugation at 1500 rpm for 5 min at 4°C. The cells pellet was lysed in ice-cold Insect Cell Lysis Buffer (Cat. No. 21425A) containing reconstituted Protease Inhibitor Cocktail (Cat. No. 21426Z) for 45 min on ice. The lysate was cleared from cellular debris by centrifugation at 14000 rpm for 1h at 4°C. The clarified lysate, which contain the recombinant protein, was then incubated with pre-washed GST-agarose beads for 18h at 4°C (10:1 ratio insect cell lysate: GST-agarose beads). After the incubation time the slurry beads were washed three times with 5 bead volumes of PBS 1X for 20 min at 4°C and centrifuged at 1500 rpm for 5 min to sediment the matrix. SDS–page was performed to determine the binding capacity of the glutathione beads using the supernatant fractions as a control and to quantify the amount of GST fusion protein that bound to the matrix by Coomassie blue-staining of the poliacrilammide gel.

### Caspase 8 in vitro cleavage assay

In vitro Caspase 8 cleavage assay was performed in a cell-free system by using Caspase 8 assay buffer (50 mM HEPES pH 7.4, 100 mM NaCl, 0.1% CHAPS, 10 mM DTT, 1 mM EDTA, 10% glycerol). GST-Us11 recombinant protein was incubated with GST-Caspase 8 recombinant protein in Caspase 8 cleavage buffer and collected at different time points (45, 60, 75, 90, 120 and 180 minutes). After the incubation time, the caspase 8 cleavage was analyzed.

### Transient transfection

293T cells were transiently transfected with pUs11 and pUs12 plasmids, separately. The Us11 and Us12 plasmids were constructed as described previously^35^. pcDNA 3.1 (Invitrogen) was used as a transfection control. Briefly, 24h prior to transfection a total of 3×10^6^ cells were seeded in 6-well plates in DMEM medium (Lonza) supplemented with 10% FBS (Euroclone). 1.5 μg of total DNA representing the plasmids described above was incubated with Lipofectamine Reagent Plus (Invitrogen) and in OptiMEM medium (Gibco) according to the manufacturer's instructions. The DNA-Lipofectamine mixture was then added to cultured cells and incubated for 4h at 37°C. The medium was then replaced with OptiMEM supplemented with 10% FBS and cells were incubated for 72h at 37°C under 5% CO_2_. The cells were then collected and processed for western blot analysis. THP-1 cells were transiently transfected with pUs11 and pUs12 plasmids by TransIT-2020 Transfection Reagent (Mirus Bio LLC, Madison, WI) according to the manufacturer’s instructions. Approximately 18-24 h before transfection, the cells were seeded at a density of 4×10^5^ cell/mL in a 12-well plate. Then, 2 μg of total DNA were complexed to TransIT-2020 reagent for 30 minutes in OptiMEM medium and subsequently, the TransIT-2020Reagent: DNA complexes was added dropwise to wells. The cells were incubated for 48 h and harvested for western blot analysis.

### Knockdown of Caspase-8 by siRNA

Knockdown of Caspase-8 was performed using a pool of chemically synthesized specific small siRNAs from Quiagen (FlexiTube GeneSolution GS841 for CASP8; GeneGlobe Id: GS841; Catalog Number: 1027416). Briefly, HEp-2 (2.5 X 10 ^5^ cell/well) were seeded onto 6 well plates for 24 h. Then, 300 nM of each siRNAs targeting different region of caspase-8 (siRNA CASP8) or negative control siRNA (siRNA NT) were transfected on HEp-2 cells by Lipofectamine RNAiMAX Transfection Reagent (Invitrogen) according to the manufacturer's instructions. The cells were then infected according to the experimental procedure.

### Viral DNA extraction and qPCR

The cell pellets were lysed using TRIzol (Life Technologies, CA, United States), according to the manufacturer’s instruction. The DNA solutions were extracted with phenol-chloroform and precipitated from the interphase and organic phase with 100% ethanol. The DNA pellet was washed twice with 0.1 M sodium citrate in 10% ethanol and dissolved with 8 mM NaOH. The concentration of DNA was determined by fluorometer analysis with the Qubit dsDNA HS (High Sensitivity) Assay Kit according to the manufacturer’s instruction. Quantitative Real-Time PCR was performed in a Cepheid Smart Cycler II System (Cepheid Europe, Maurens-Scopont, France), using a specific TaqMan probe. Total cellular DNA (1µg) was mixed with 0.5μM of each forward and reverse primers, 1µM of TaqMan probe, 1 µM of dNTP mix, NH_4_ reaction buffer 1X, 2mM of MgCl_2_, and 5U/µL of thermostable DNA polymerase BIOTAQTM (BIO-21040 Bioline) in a total volume of 25µL. The oligonucleotide primer pairs were as follows: HSV-1 Fw 5’-catcaccgacccggagagggac; HSV-1 Rev 5’gggccaggcgcttgttggtgta, HSV-1 TaqMan probe 5’-6FAM-ccgccgaactgagcagacacccgcgc-TAMRA, (6FAM is 6carboxyfluorescein and TAMRA is 6-carboxytetramethylrhodamine). The amplification was performed following specific steps (10 min at 95 °C, 30 s at 95 °C for 40 cycles, 30 s at 55 °C, and 30 s at 72 °C, 5 min at 72 °C) and a negative sample was used as amplification control for each run. The relative quantification of HSV-1 DNA was generated by comparative Ct method using GAPDH as a housekeeping gene.

### Quantification and Statistical Analysis

Data are expressed as results of the mean ± SD of three independent experiments. For data analysis, the Graphpad Prism 6 software (GraphPad Software, San Diego, CA, USA) was used. Student's t-test and One-way ANOVA were used for statistical analysis to compare different conditions. The asterisks (*, **, *** and ****) indicate the significance of p-values less than 0.05, 0.01, 0.001 and 0.0001, respectively. Immunofluorescence images were acquired using the Leica SP5 microscope. Quantitative densitometry analysis of immunoblot band intensities was performed by using the TINA software (version 2.10, Raytest, Straubenhardt, Germany).

## Supplementary Information

Supplementary Information 1.

Supplementary Information 2.

Supplementary Information 3.

Supplementary Information 4.

Supplementary Information 5.

Supplementary Information 6.

Supplementary Information 7.

## Data Availability

All data generated during this study are included in this published article (and its supplementary information files).
