# Supplementary material for: Direct cleavage of caspase-8 by herpes simplex virus 1 tegument protein US11
Source: Sci Rep. 2022 Jul 19;12:12317. doi: 10.1038/s41598-022-15942-9 (PMC9296525; doi:10.1038/s41598-022-15942-9)
Supplement: Supplementary file 1 — Supplementary Information 1. [file 41598_2022_15942_MOESM1_ESM.pdf]

Supplementary Information 1

Direct cleavage of Caspase-8 by Herpes Simplex Virus 1 Tegument Protein US11

Maria Musarra-Pizzo[1]*, Rosamaria Pennisi[1], Daniele Lombardo[2], Tania Velletri[3] and Maria Teresa Sciortino[1]*

[1]Department of Chemical, Biological, Pharmaceutical and Environmental Sciences, University of Messina, Messina, Italy, 98168, Europe.

[2]Division of Clinical and Molecular Hepatology, University Hospital 'G. Martino' of Messina, Messina, 98124, Italy

[3]IFOM-Cogentech Società Benefit srl; via Adamello 16, 20139 Milan, Italy-Local Unit: Scientific and Technological Park of Sicily- 95121 Catania, Italy.

*Corresponding authors: Maria Teresa Sciortino and Maria Musarra Pizzo

Supplementary figure S1.

Original image of Figure 1b

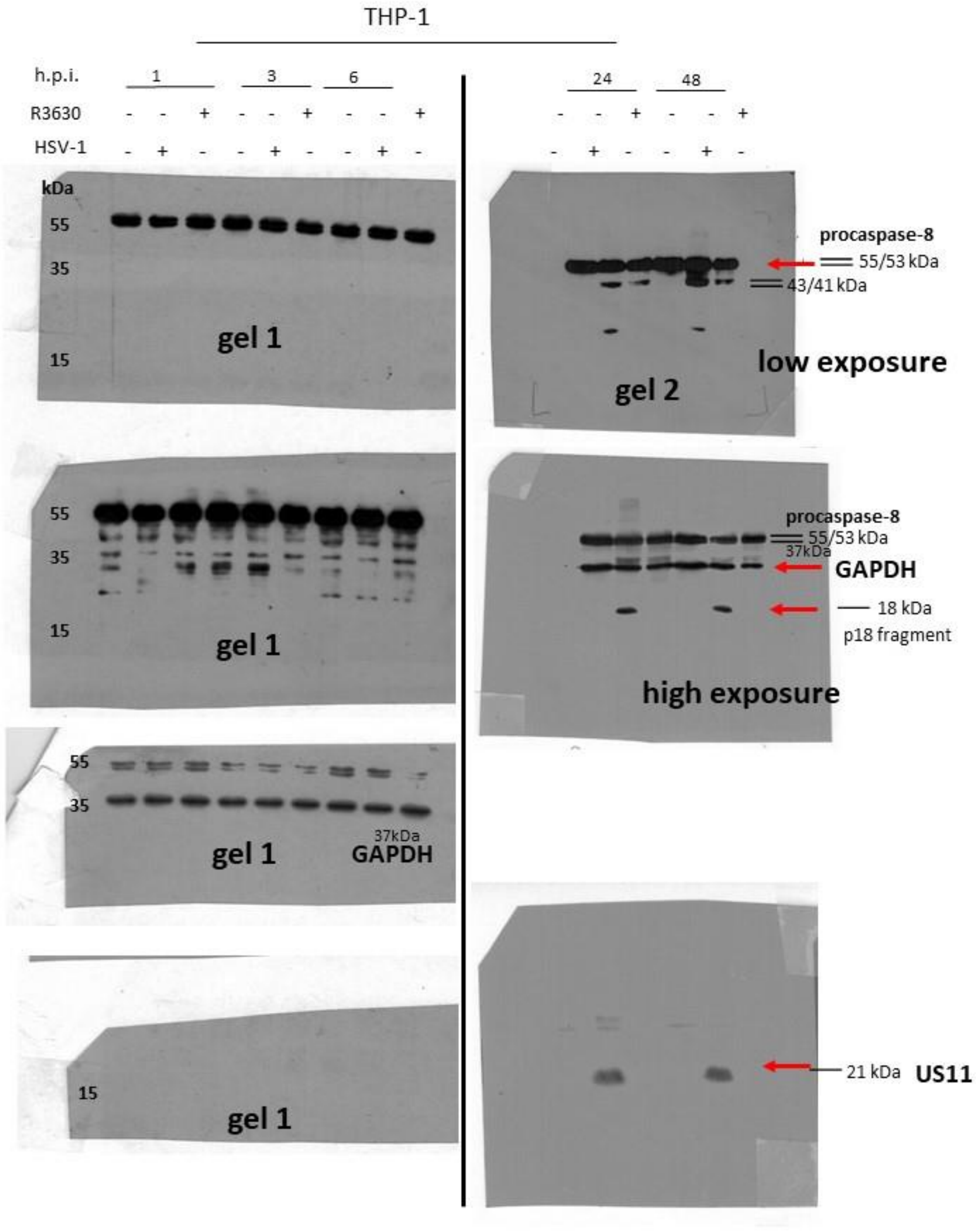

Figure S1. b) Cleavage and activation of caspase-8 during HSV-1 replication in THP-1 and effect of Us11 deletion. Time course analysis of caspase-8 in THP-1 cells infected or not with HSV-1 and R3630 (ΔUs11/Us12) viruses. The grouping blots are cropped from two different gels (gel 1 and gel 2), as displayed in figure. The expression of each protein at 1, 3, 6, 24h and 48h was simultaneously revealed. Multiple exposures of cleavage of caspase 8 have been shown. To improve the clarity and conciseness of the presentation, the figure was presented as follow: procaspase-8 full length and p43/41 from low exposure; p18 fragment from high exposure. Both derived from same gels: 1gel for 1hr,3hr and 6hr and 2gel for 24hr and 48hr. Arrowheads indicate bands corresponding to target proteins

Supplementary figure S1.

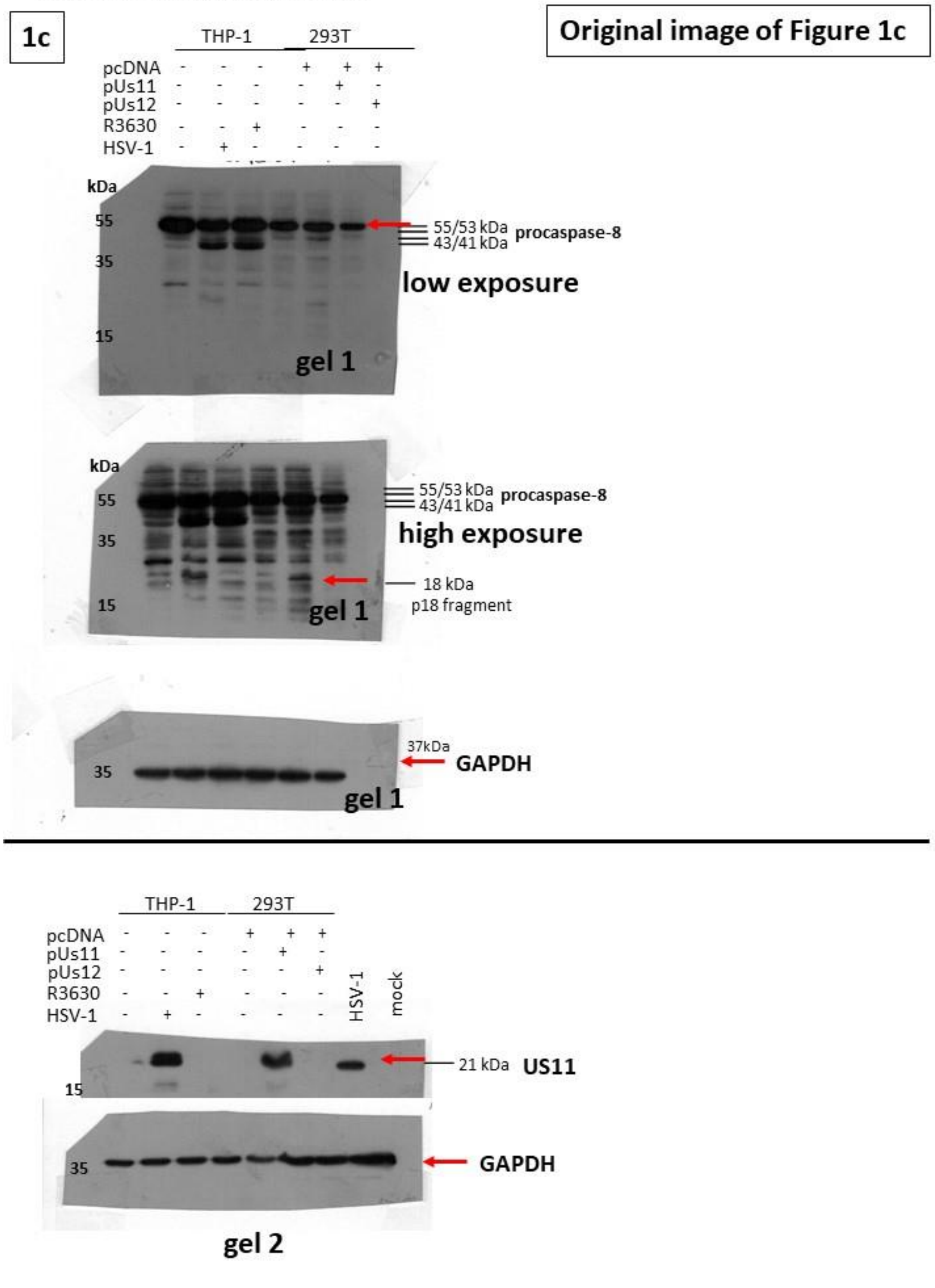

Figure S1. c) Cleavage and activation of caspase-8 during HSV-1 replication in THP-1 and effect of Us11 deletion. Analysis of caspase-8 cleavages in THP-1 cells infected or not with HSV-1 or R3630 (ΔUs11/Us12) for 24h and 293T cells transfected with pUs11 and pUs12 plasmids and collected at 72h post-transfection. Multiple exposures of cleavage of caspase 8 have been shown. To improve the clarity and conciseness of the

presentation, the figure was presented as follow: procaspase-8 full length and p43/41 from low exposure; p18 fragment from high exposure. The grouping blots are cropped from two different gels (gel 1 and gel 2), as displayed in figure. Arrowheads indicate bands corresponding to target proteins

Supplementary figure S1.

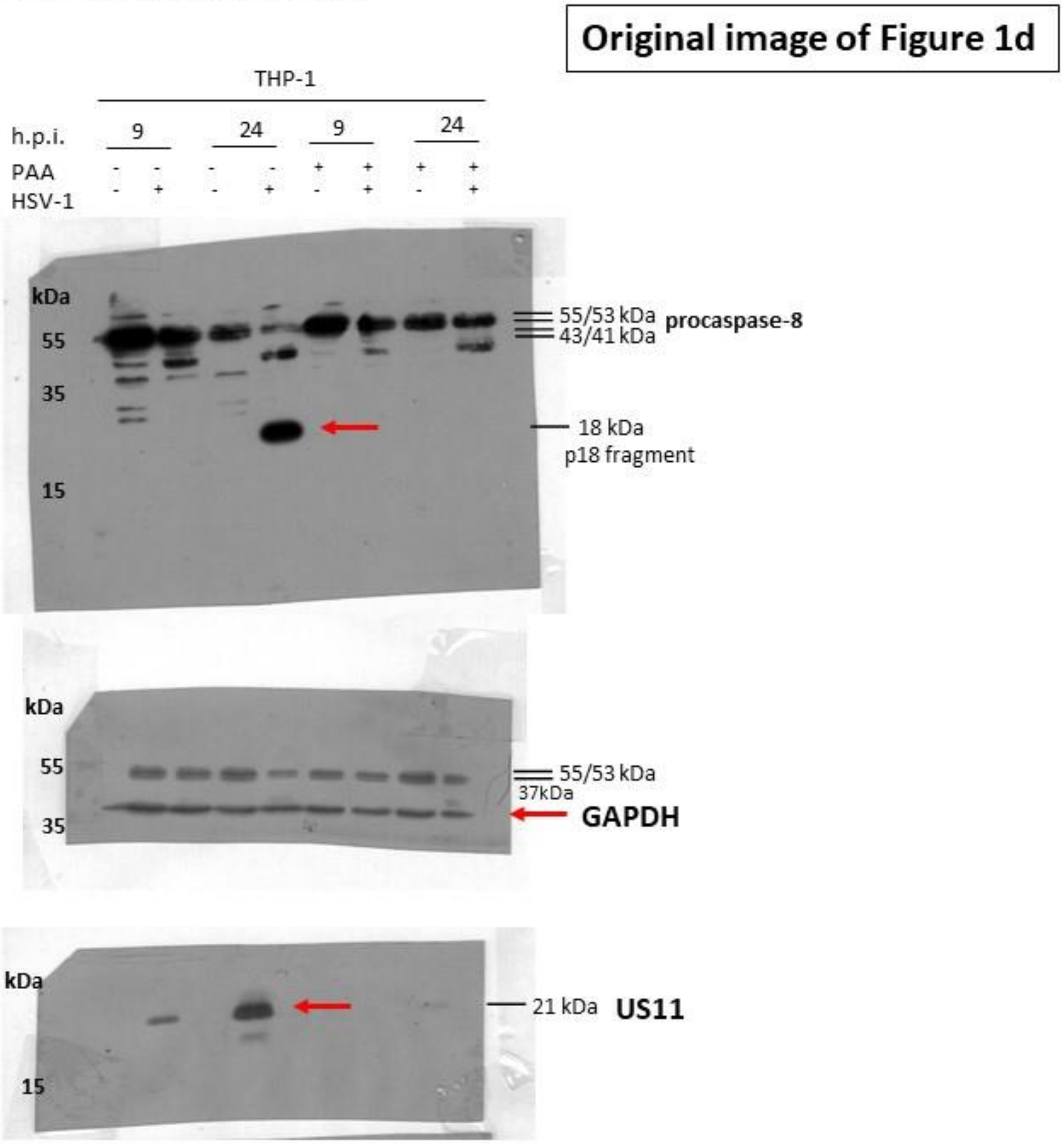

Figure S1. d) Cleavage and activation of caspase-8 during HSV-1 replication in THP-1 and effect of Us11 deletion. Analyses of caspase-8 cleavage in THP-1 cells infected with HSV-1 and treated or not with PAA (300 μg/ml). To improve the clarity and conciseness of the presentation, the figure was presented as cropping parts of the same gel first blotted with anti-caspase-8 antibody and then with anti-GAPDH. Arrowheads indicate bands corresponding to target proteins

Supplementary figure S1.

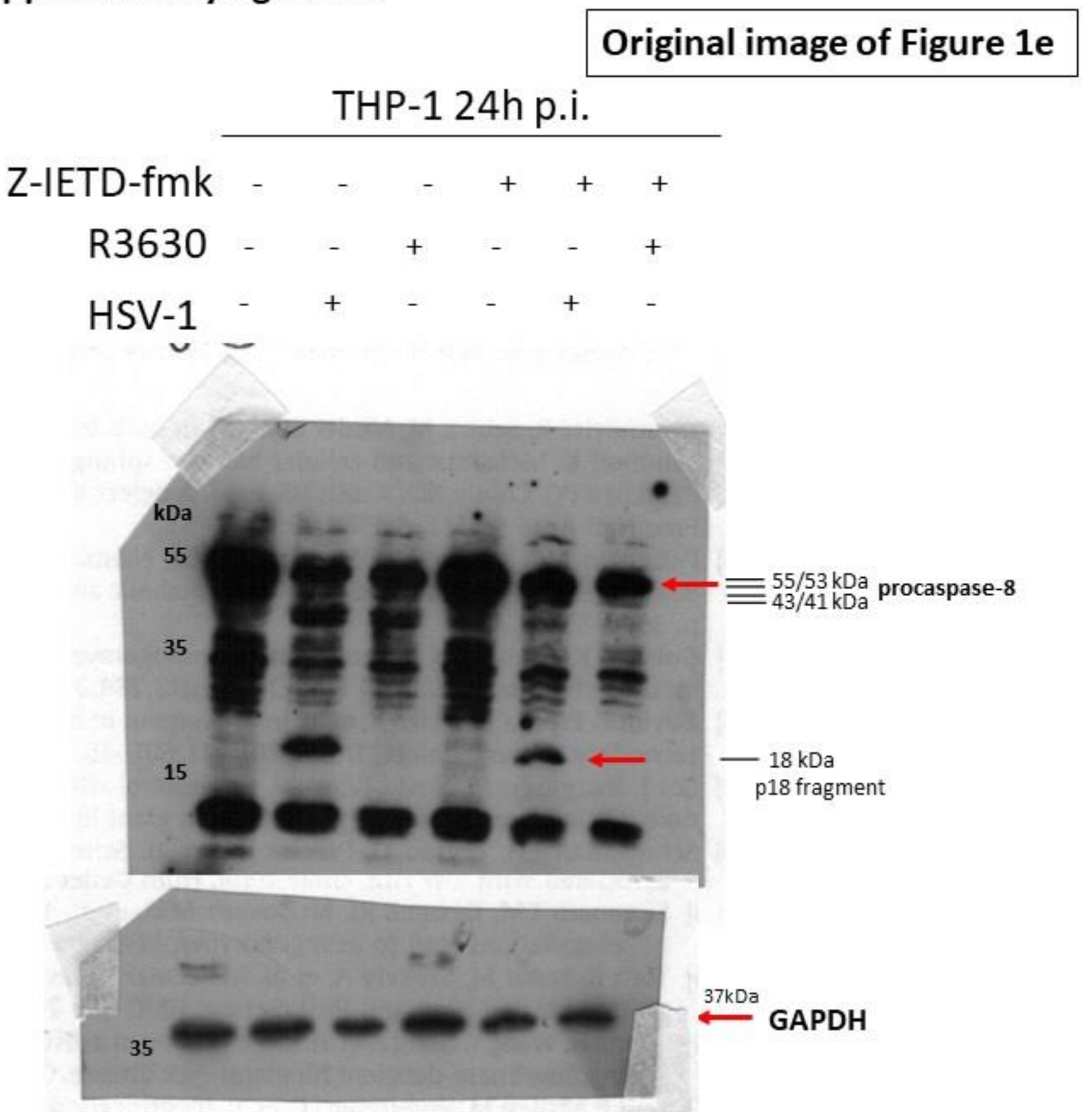

Figure S1. e) Cleavage and activation of caspase-8 during HSV-1 replication in THP-1 and effect of Us11 deletion. Analysis of caspase-8 cleavage in THP-1 cells infected with HSV-1 or R3630 (ΔUs11/Us12) and treated or not with the caspase-8 inhibitor z-IETD-fmk (100 µM); The full-length (p55/53) and cleaved form (p43/41 and p18) of caspase-8 was detected by using a specific antibody directed to the p18 subunit (ALX-804-242-12F5). GAPDH was used as loading control. To improve the clarity and conciseness of the presentation, the figure was presented as cropping parts of the same gel first blotted with anti-caspase-8 antibody and then with anti-GAPDH. Arrowheads indicate bands corresponding to target proteins.

Supplementary figure S1.

0 h.p.i.

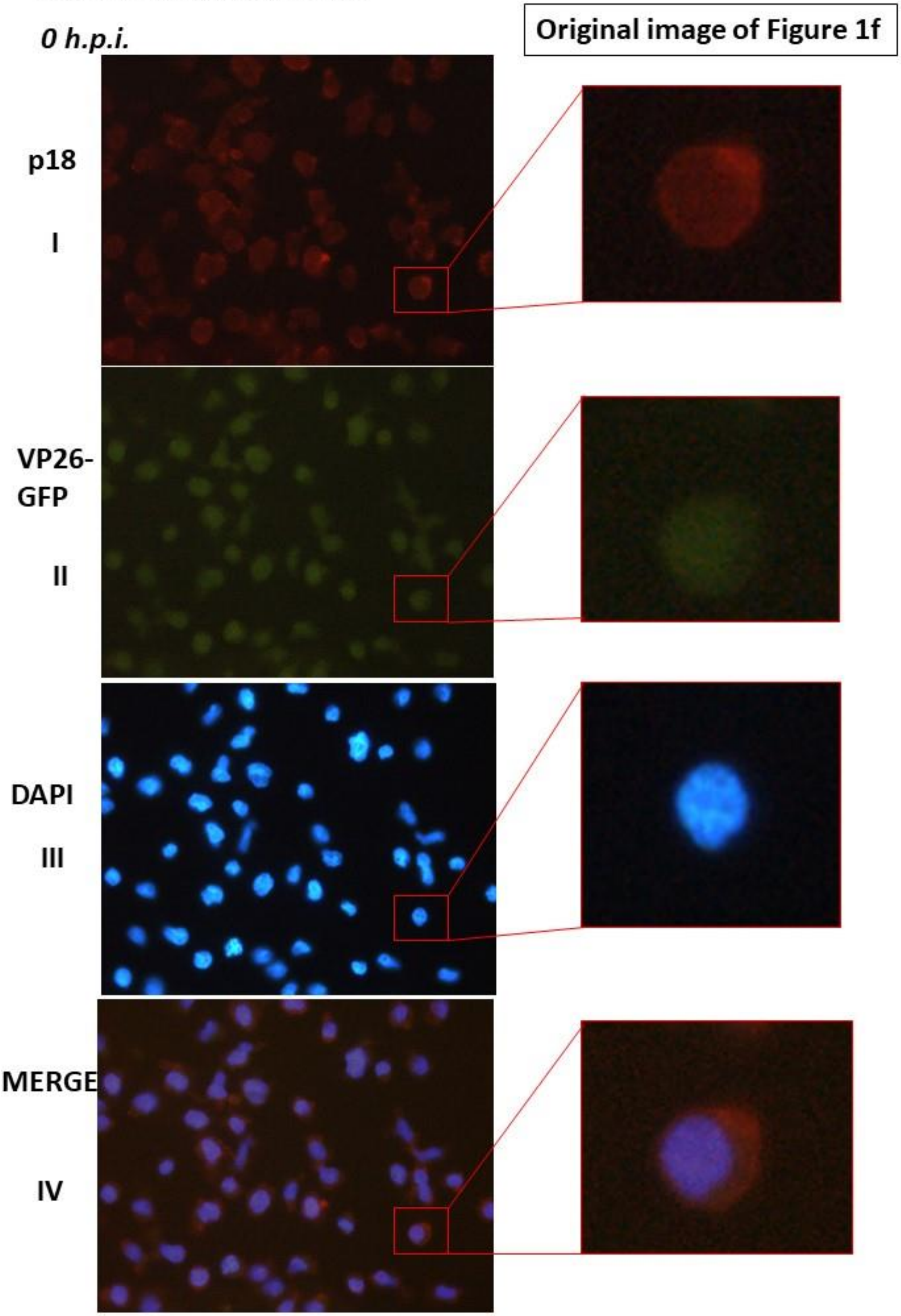

Supplementary figure S1.

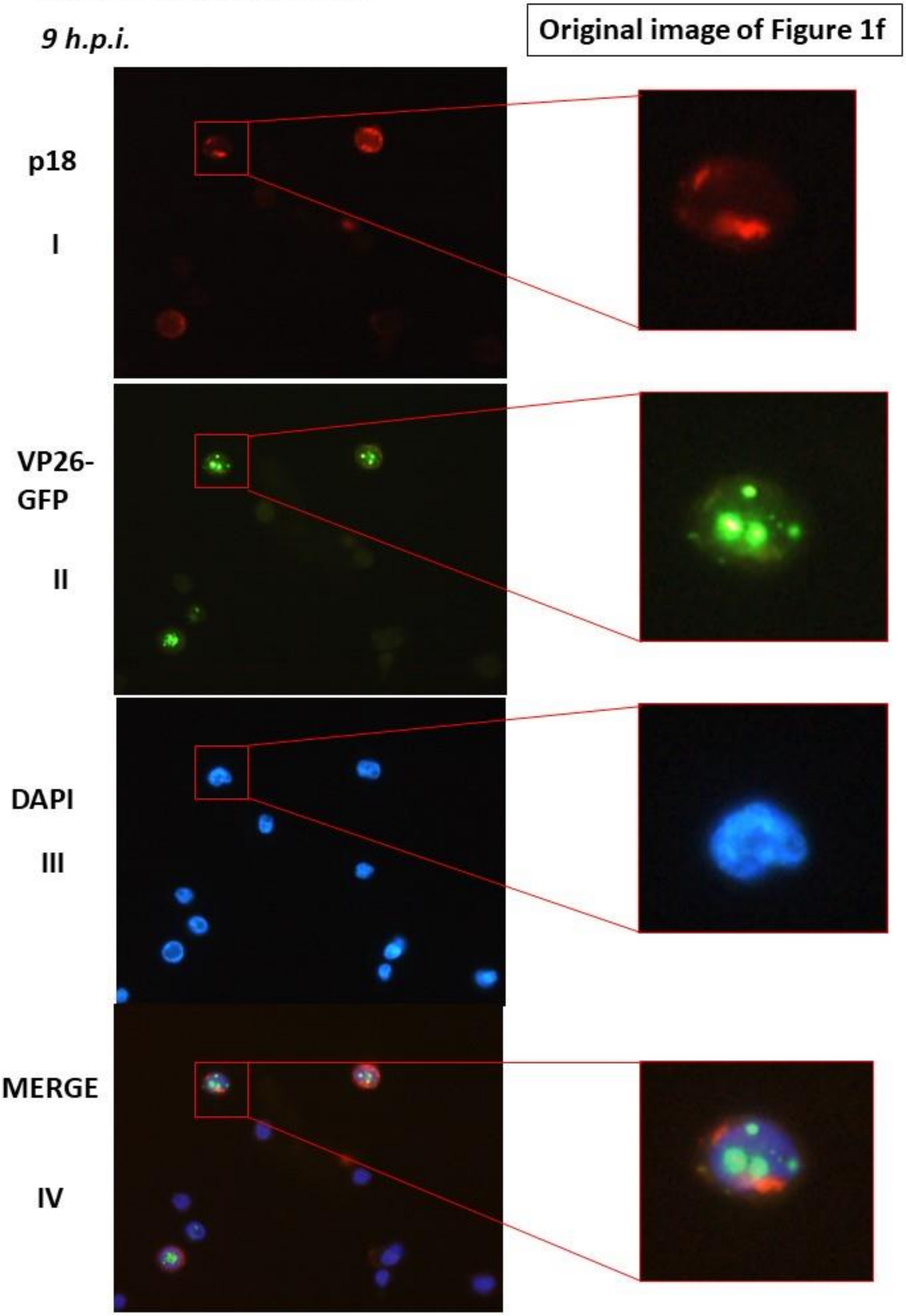

Supplementary figure S1.

24 h.p.i.

Original image of Figure 1f

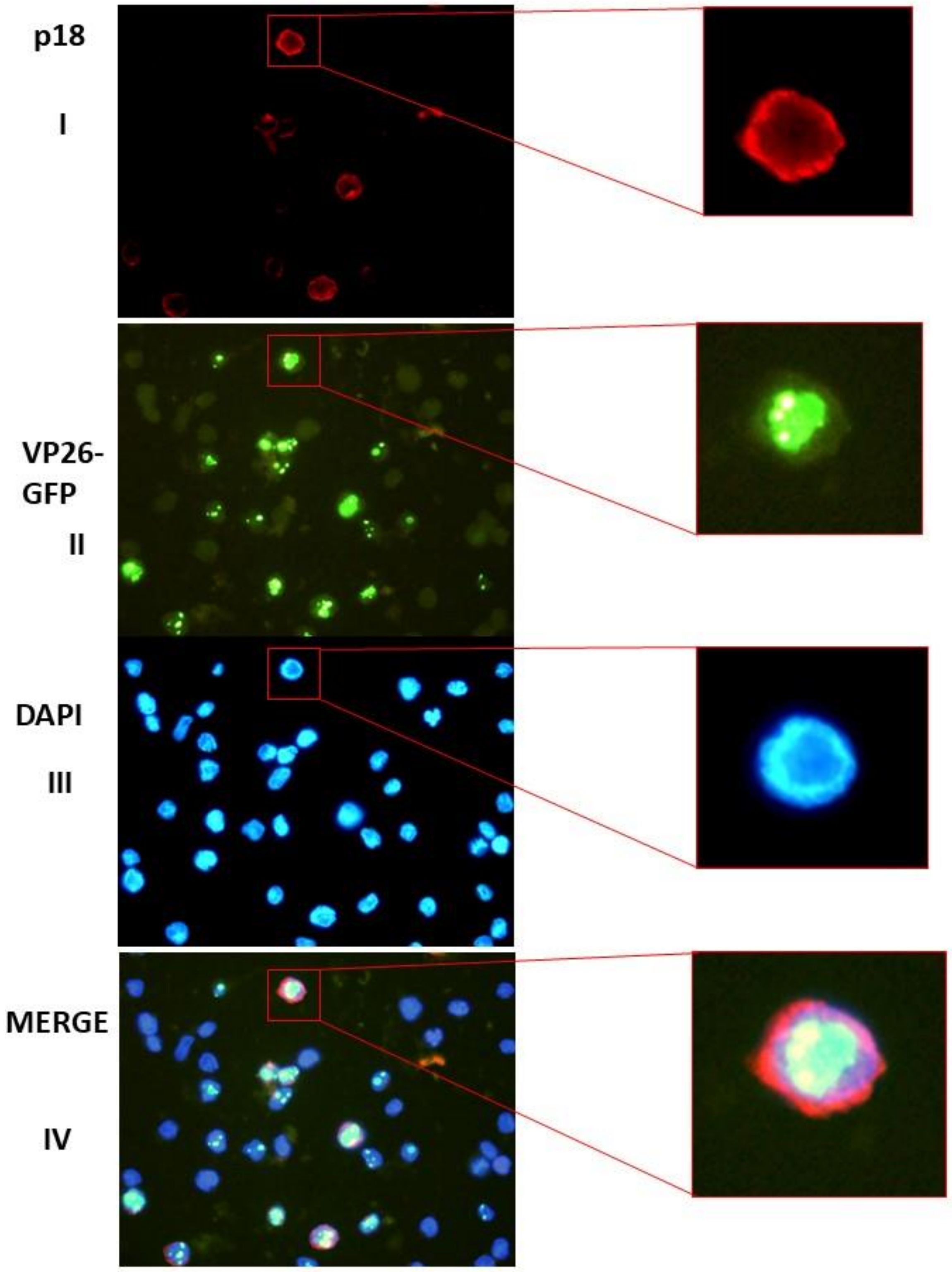

Supplementary figure S1.

36 h.p.i.

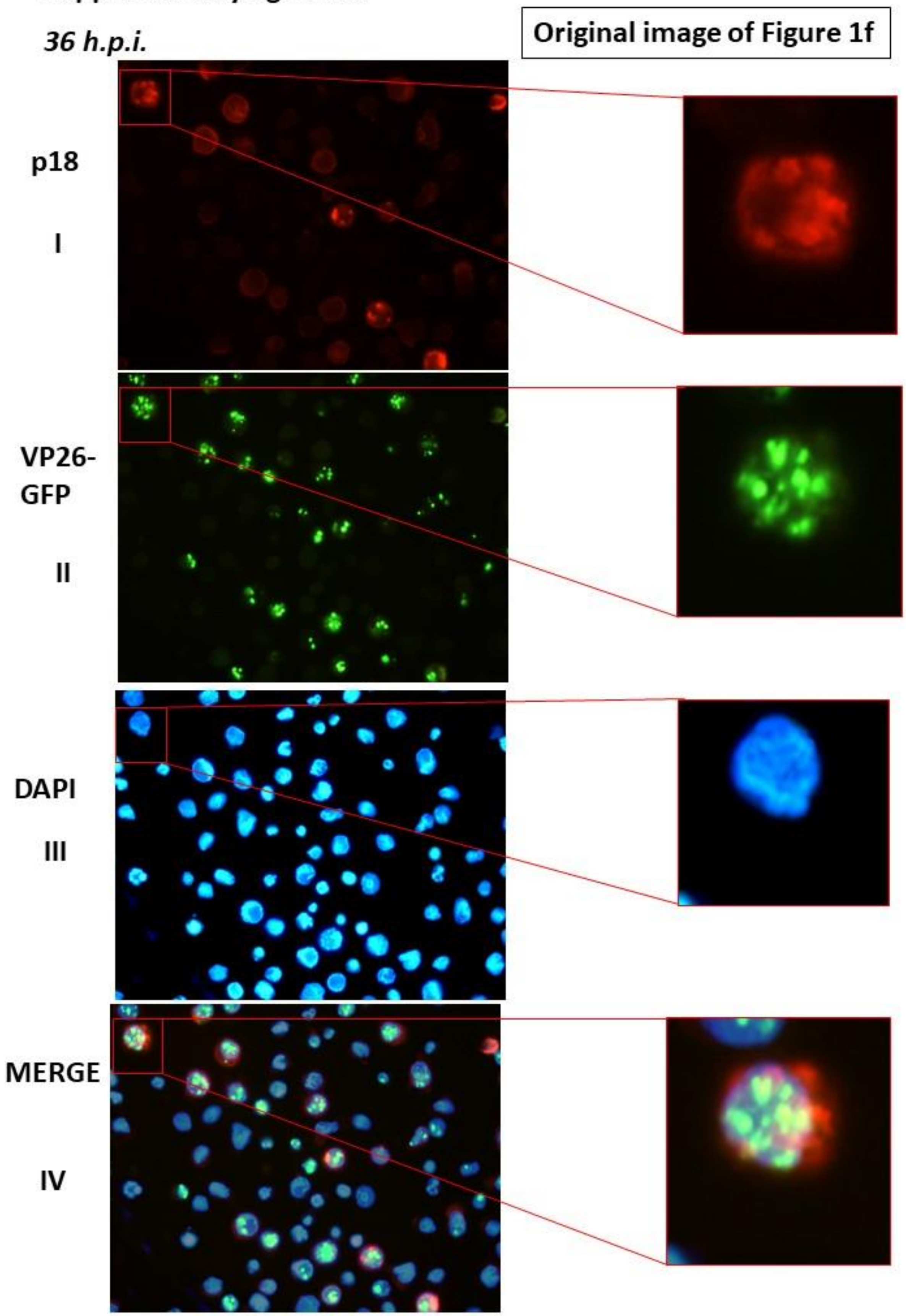

Figure S1. Cleavage and activation of caspase-8 during HSV-1 replication in THP-1 and effect of Us11 deletion. f) fluorescence microscope analysis of p18 fragment in THP-1 infected with HSV-1-VP26GFP. The cells were infected or not with HSV-1-VP26GFP and stained with anti-p18 antibody in red (I). The green dots are representative of autofluorescent VP26GFP protein (II). Hoechst was used to stain the nuclei (III); the IV column represent the merged images; Magnification of images x63. The original images of the fluorescence microscope analysis were reported and the red boxes show the detail presented in the manuscript.

Supplementary figure S1.

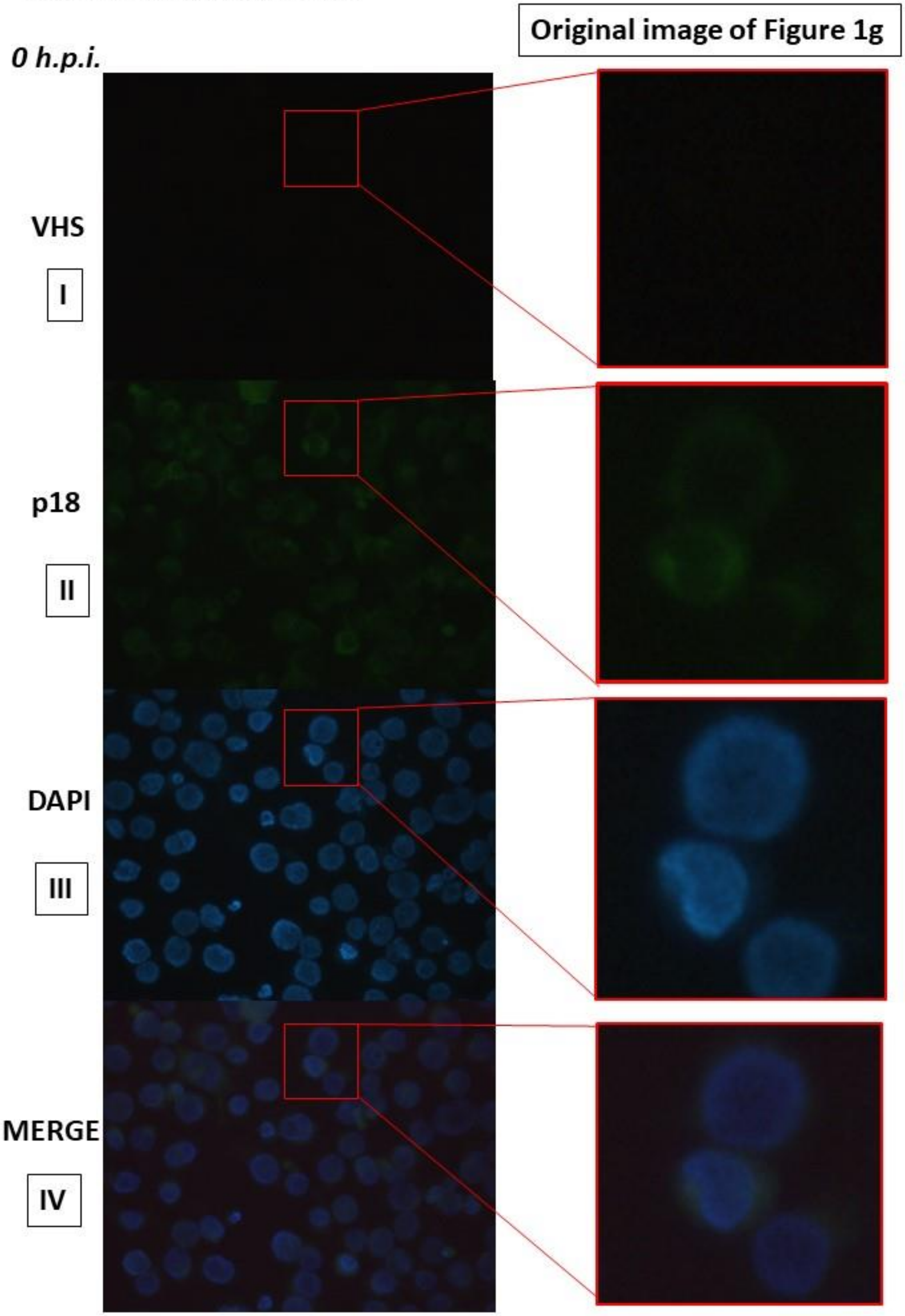

Supplementary figure S1.

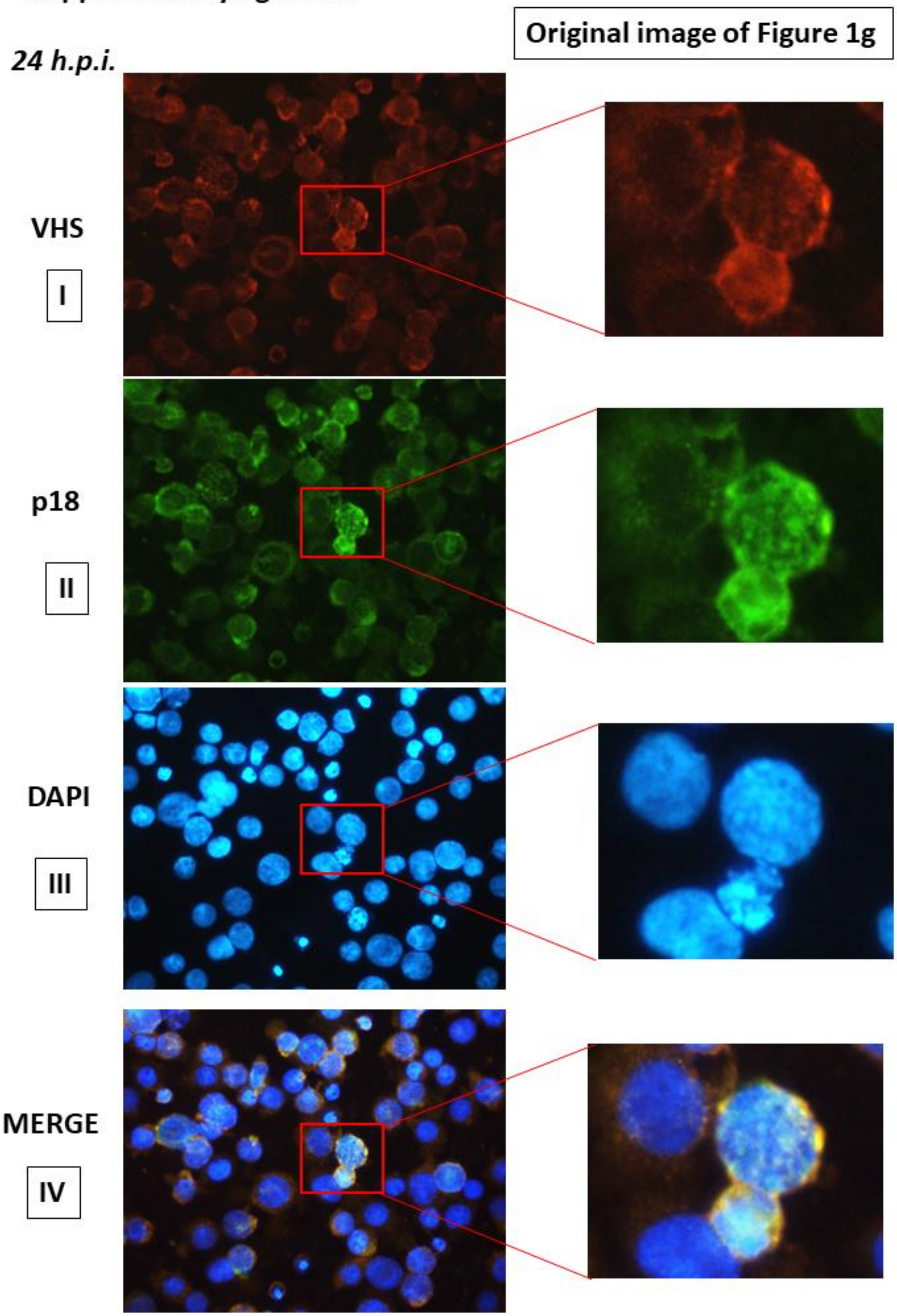

Figure S1. g) Cleavage and activation of caspase-8 during HSV-1 replication in THP-1 and effect of Us11 deletion. Co-localization of p18 with the late gene product VHS. THP-1 cells were infected or not with HSV-1 and stained with both anti-p18 (II) and anti-VHS antibodies (I). Hoechst was used to stain the nuclei (III); the IV column represent the merged images. Magnification of images x63. The original images of the fluorescence microscope analysis were reported and the red boxes show the detail presented in the manuscript.
