# Supplementary material for: Direct cleavage of caspase-8 by herpes simplex virus 1 tegument protein US11
Source: Sci Rep. 2022 Jul 19;12:12317. doi: 10.1038/s41598-022-15942-9 (PMC9296525; doi:10.1038/s41598-022-15942-9)
Supplement: Supplementary file 2 — Supplementary Information 2. [file 41598_2022_15942_MOESM2_ESM.pdf]

Supplementary Information 2

Direct cleavage of Caspase-8 by Herpes Simplex Virus 1 Tegument Protein US11

Maria Musarra-Pizzo[1]*, Rosamaria Pennisi[1], Daniele Lombardo[2], Tania Velletri[3] and Maria Teresa Sciortino[1]*

[1]Department of Chemical, Biological, Pharmaceutical and Environmental Sciences, University of Messina, Messina, Italy, 98168, Europe.

[2]Division of Clinical and Molecular Hepatology, University Hospital 'G. Martino' of Messina, Messina, 98124, Italy

[3]IFOM-Cogentech Società Benefit srl; via Adamello 16, 20139 Milan, Italy-Local Unit: Scientific and Technological Park of Sicily- 95121 Catania, Italy.

*Corresponding authors: Maria Teresa Sciortino and Maria Musarra Pizzo

Supplementary figure S2.

Original image of Figure 2a

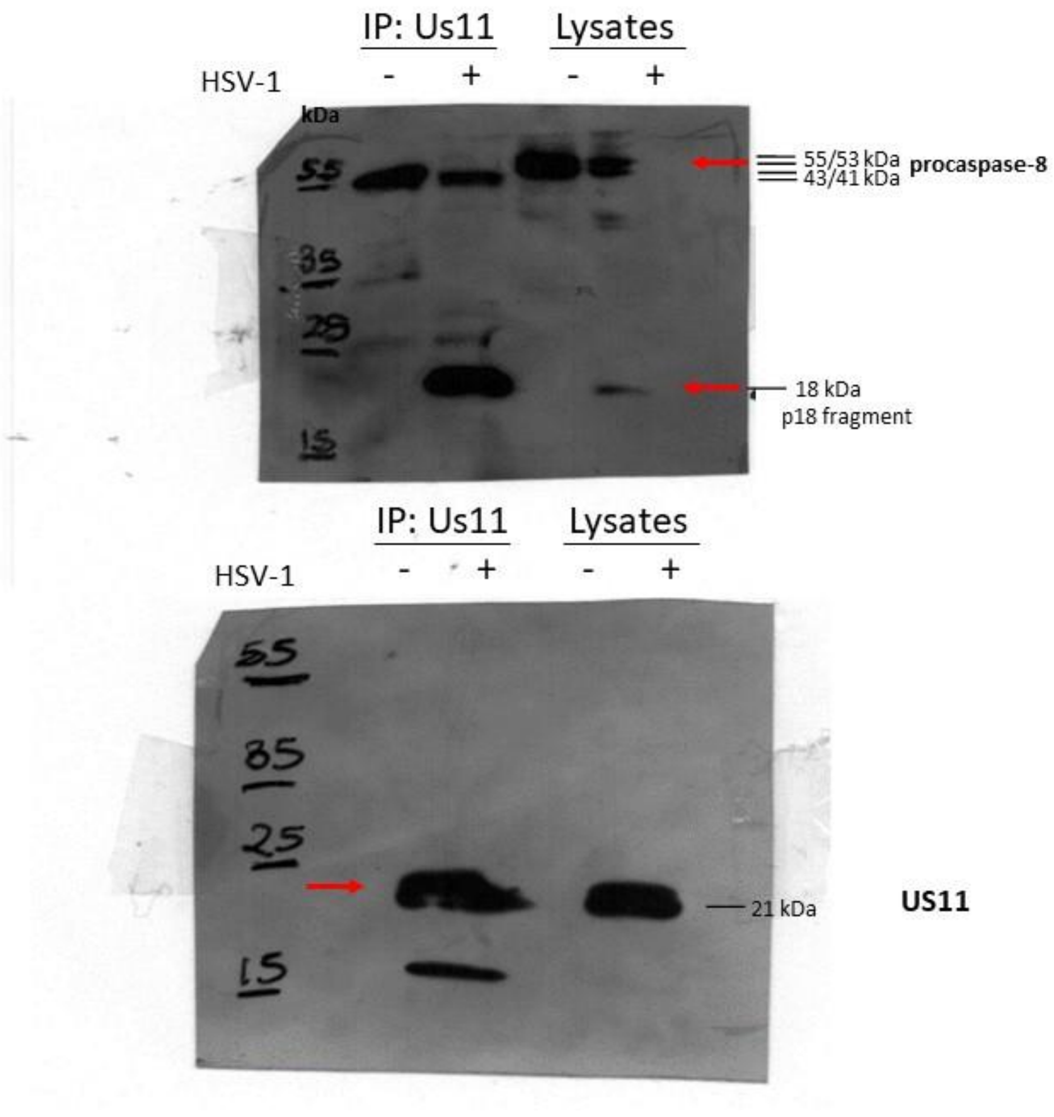

Figure S2. a) Immunoprecipitation and caspase-8 cleavage assay. THP-1 cells were infected or not with HSV-1, lysed and then incubated overnight at 4 °C with 5µl of Us11 antibody pre-adsorbed on protein-A Sepharose Beads. After overnight incubation with the extracts, complexate-beads were resolved by SDS-PAGE and transferred to nitrocellulose membranes. To improve the clarity and conciseness of the presentation, the figure was presented as cropping parts of the same gel first blotted with anti-caspase-8 antibody and then with anti-US11. Arrowheads indicate bands corresponding to target proteins.

Supplementary figure S2.

Original image of Figure 2b

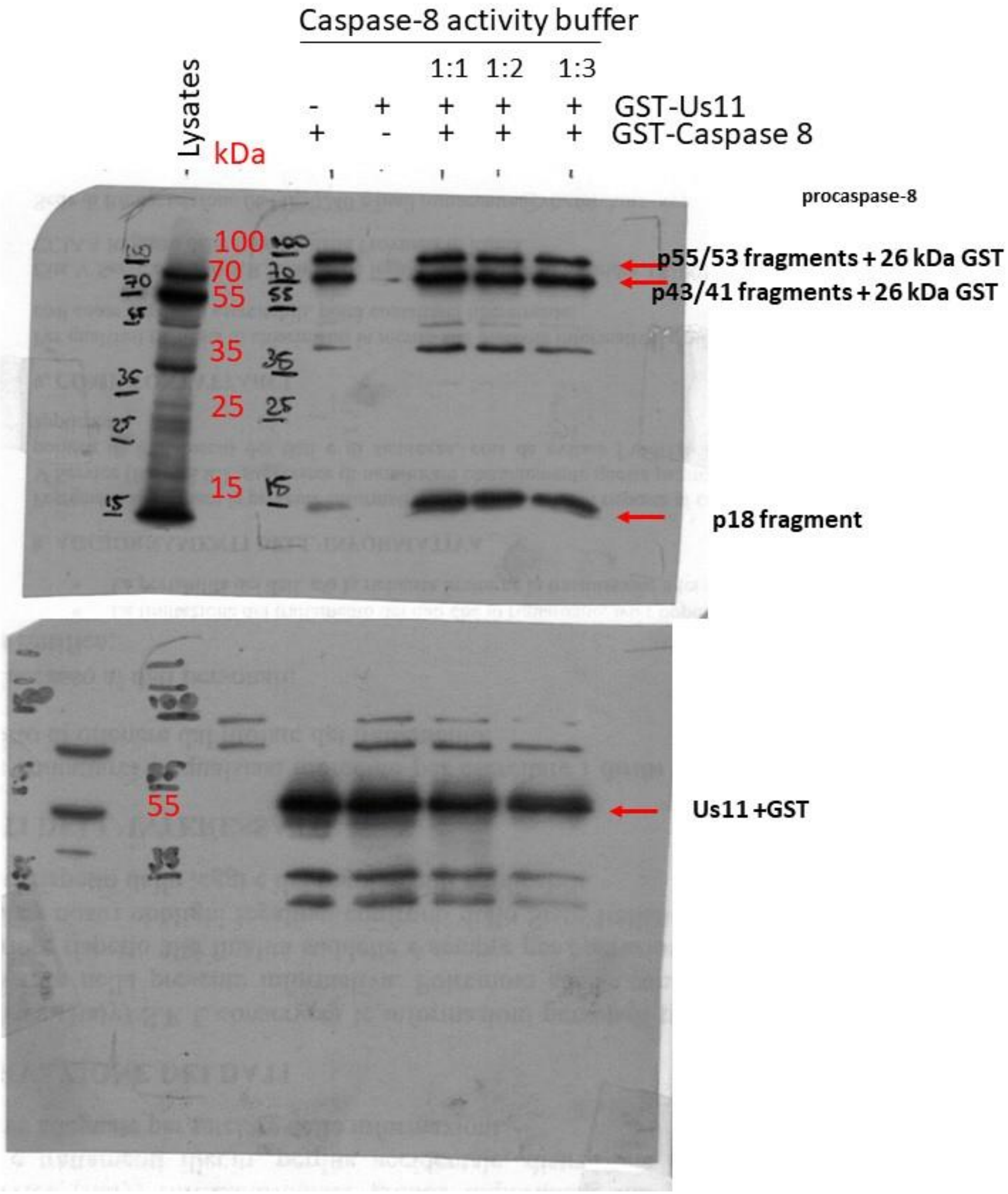

Figure S2. b) Immunoprecipitation and caspase-8 cleavage assay: GST-caspase-8 recombinant protein was incubated with a serial dilution of GST-Us11 recombinant protein (1:1, 1:2, 1:3) in caspase-8 assay buffer as

described in material and methods. After 1h incubation time, the activation of caspase 8 was verified by SDS-PAGE. the membrane was first blotted with anti-caspase-8 antibody and then with anti-US11. Arrowheads indicate bands corresponding to target proteins.

Supplementary figure S2.

Original image of Figure 2c

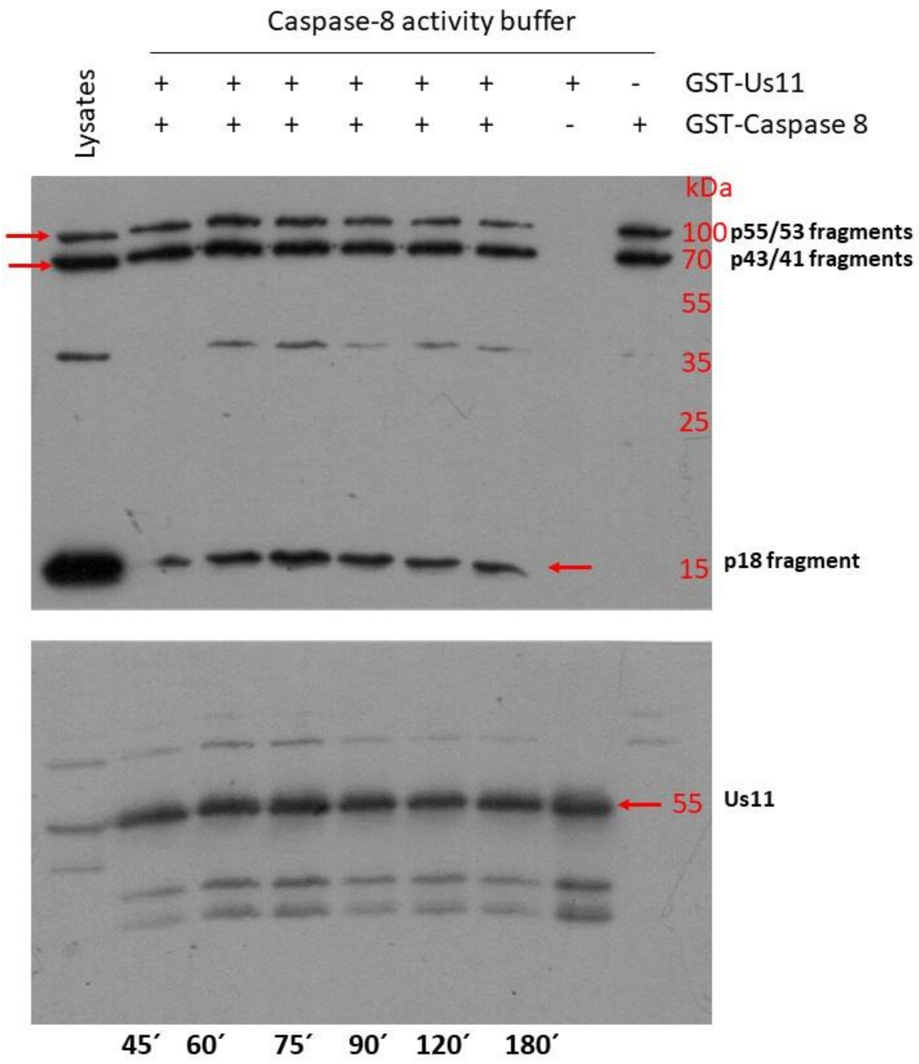

Figure S2. c) Immunoprecipitation and caspase-8 cleavage assay: GST-Caspase 8 recombinant protein was incubated with GST-Us11 recombinant protein (1:1) in caspase-8 assay buffer for a different time from 45 min up to 3h. After the incubation time, the activation of caspase 8 was verified by SDS-PAGE. The membrane was first blotted with anti-caspase-8 antibody and then with anti-US11. Arrowheads indicate bands corresponding to target proteins.
