# Supplementary material for: Direct cleavage of caspase-8 by herpes simplex virus 1 tegument protein US11
Source: Sci Rep. 2022 Jul 19;12:12317. doi: 10.1038/s41598-022-15942-9 (PMC9296525; doi:10.1038/s41598-022-15942-9)
Supplement: Supplementary file 3 — Supplementary Information 3. [file 41598_2022_15942_MOESM3_ESM.pdf]

Supplementary Information 3

Direct cleavage of Caspase-8 by Herpes Simplex Virus 1 Tegument Protein US11

Maria Musarra-Pizzo[1]*, Rosamaria Pennisi[1], Daniele Lombardo[2], Tania Velletri[3] and Maria Teresa Sciortino[1]*

[1]Department of Chemical, Biological, Pharmaceutical and Environmental Sciences, University of Messina, Messina, Italy, 98168, Europe.

[2]Division of Clinical and Molecular Hepatology, University Hospital 'G. Martino' of Messina, Messina, 98124, Italy

[3]IFOM-Cogentech Società Benefit srl; via Adamello 16, 20139 Milan, Italy-Local Unit: Scientific and Technological Park of Sicily- 95121 Catania, Italy.

*Corresponding authors: Maria Teresa Sciortino and Maria Musarra Pizzo

Supplementary figure S3.

Original image of Figure 3a

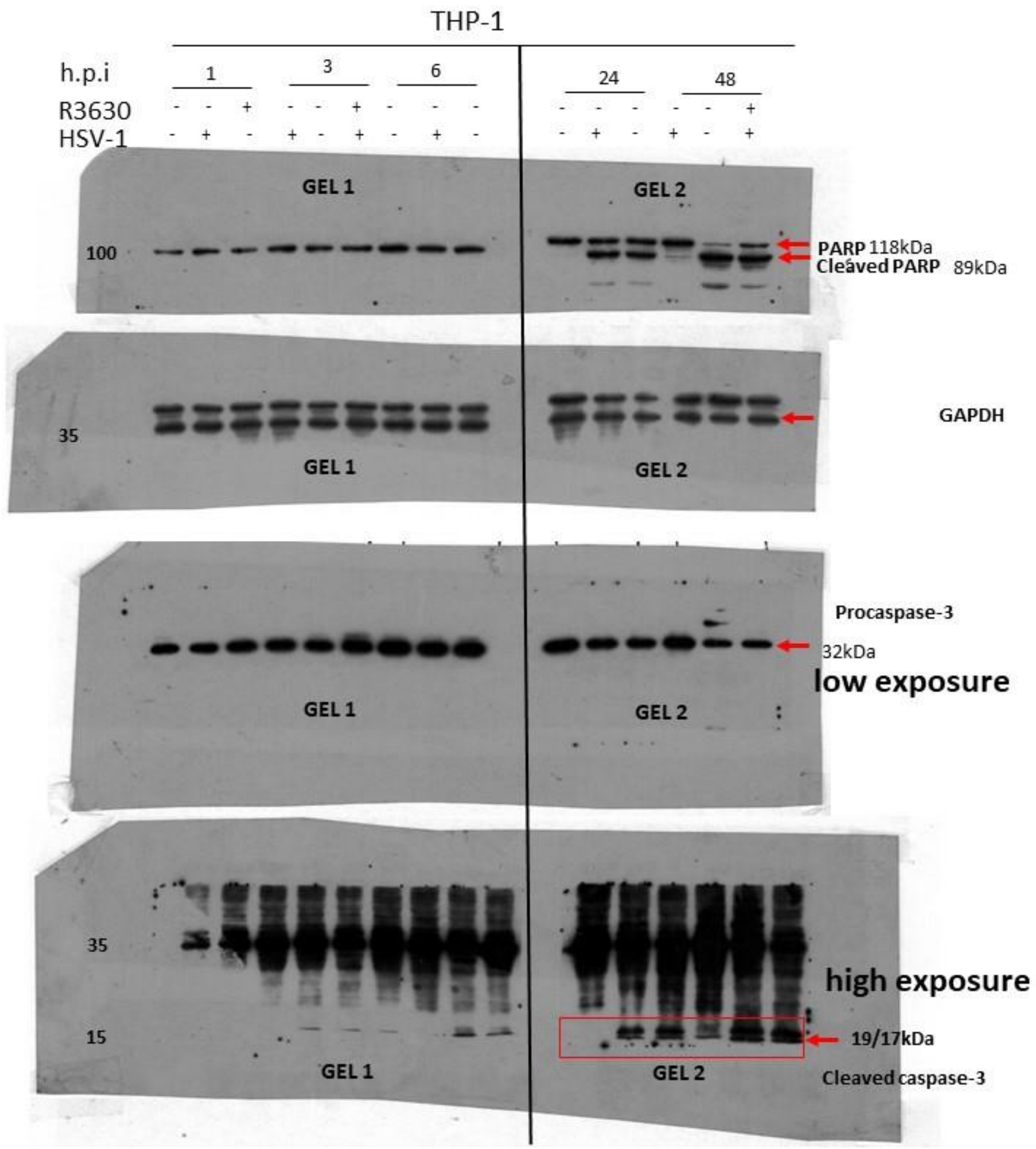

Figure S3. Analysis of apoptotic markers. **a**) Western blot analysis of PARP and caspase-3 from: THP-1 cells infected with HSV-1 or R3630 (ΔUs11/Us12) and collected at 1,3,6,24 and 48 h.p.i; The grouping blots are cropped from two different gels (GEL 1 and GEL 2,), as displayed in the figure, and exposed simultaneously.

Multiple exposures of caspase 3 have been shown and reported as cropped image to improve the clarity and conciseness of the presentation. Arrowheads indicate bands corresponding to target proteins. The boxes indicate the cleaved fragment of caspase3.

Supplementary figure S3.

Original image of Figure 3b

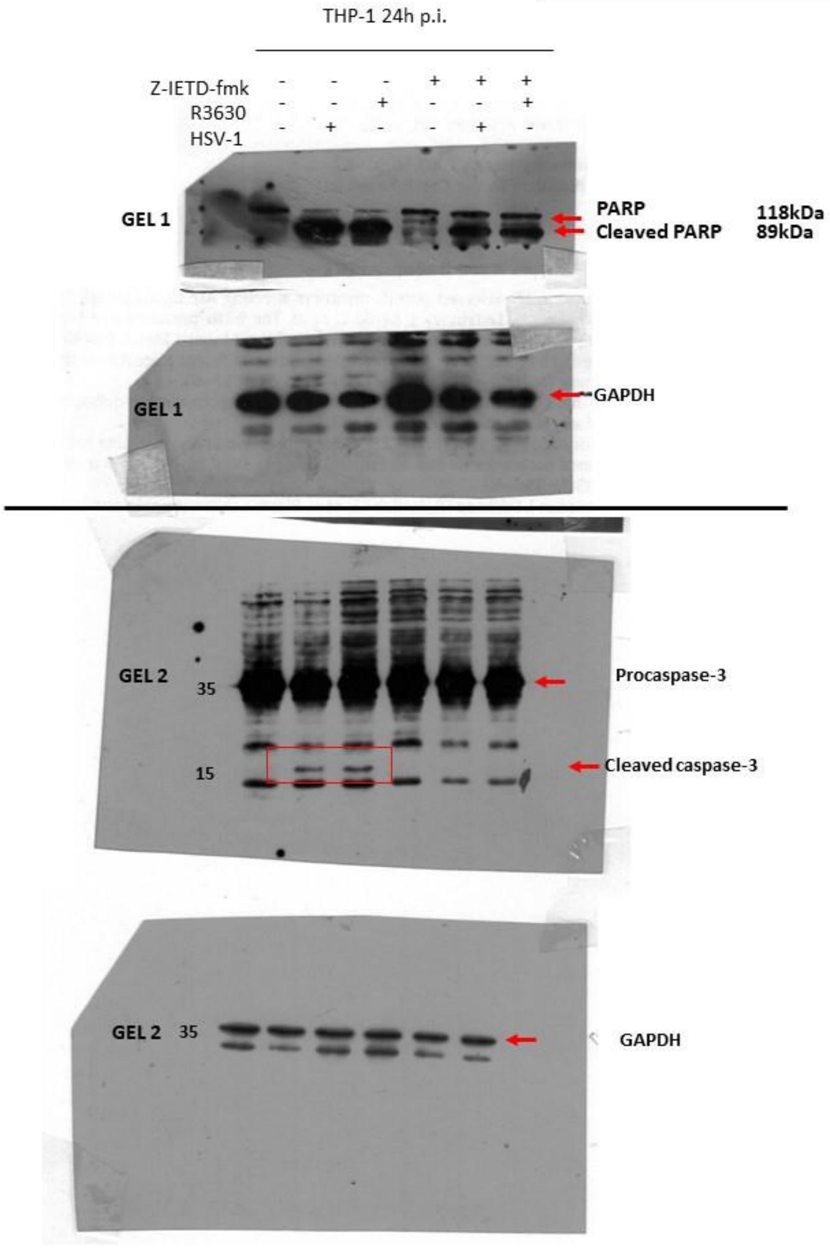

Figure S3. Analysis of apoptotic markers. b) THP-1 cells infected with HSV-1 or R3630 (ΔUs11/Us12) and treated or not with the caspase-8 inhibitor z-IETD-fmk (100 µM); The grouping blots are cropped from two different gels (GEL 1 and GEL 2,), as displayed in the figure. Arrowheads indicate bands corresponding to target proteins. The boxes indicate the cleaved fragment of caspase3.

Supplementary figure S3.

Original image of Figure 3c

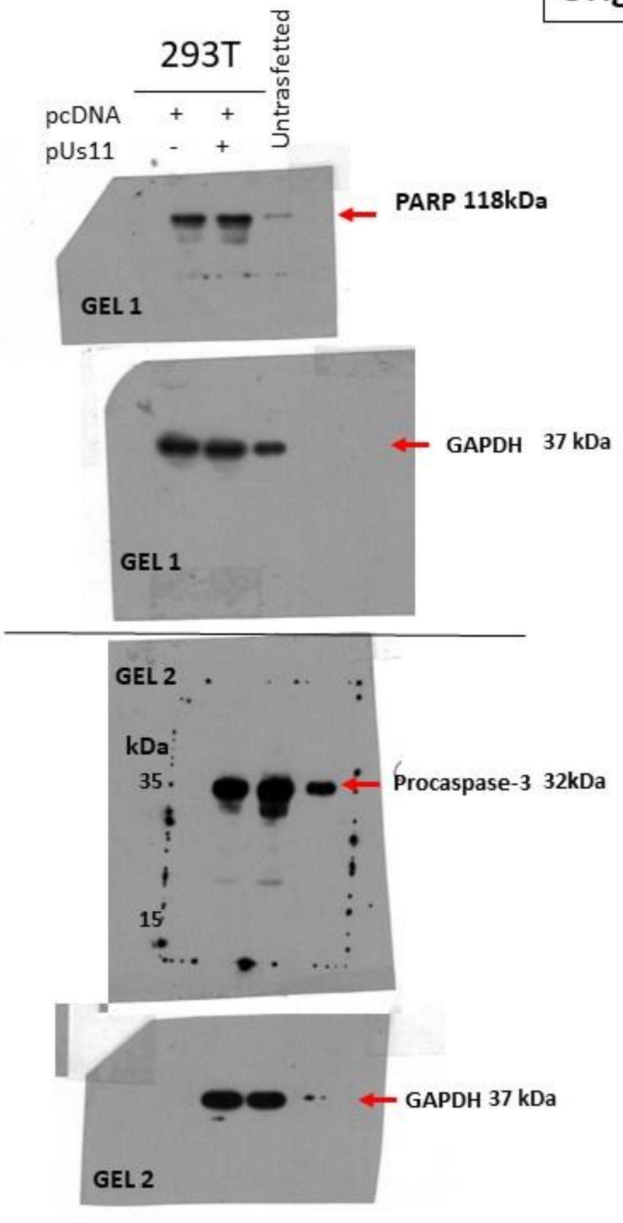

Figure S3. Analysis of apoptotic markers. c) THP-1 cells transfected with pUs11 and collected 72h post transfection. The membranes were probed with an antibody directed to caspase-3 and PARP. GAPDH was used as a loading control. The grouping blots are cropped from two different gels (GEL 1 and GEL 2,) as displayed in the figure. The membrane 1 was cut and probed with PARP and GAPDH, separately. Instead, the membrane 2 was probed first with caspase-3 antibody and then with GAPDH. Arrowheads indicate bands corresponding to target proteins.

Supplementary figure S3.

Original image of Figure 3d

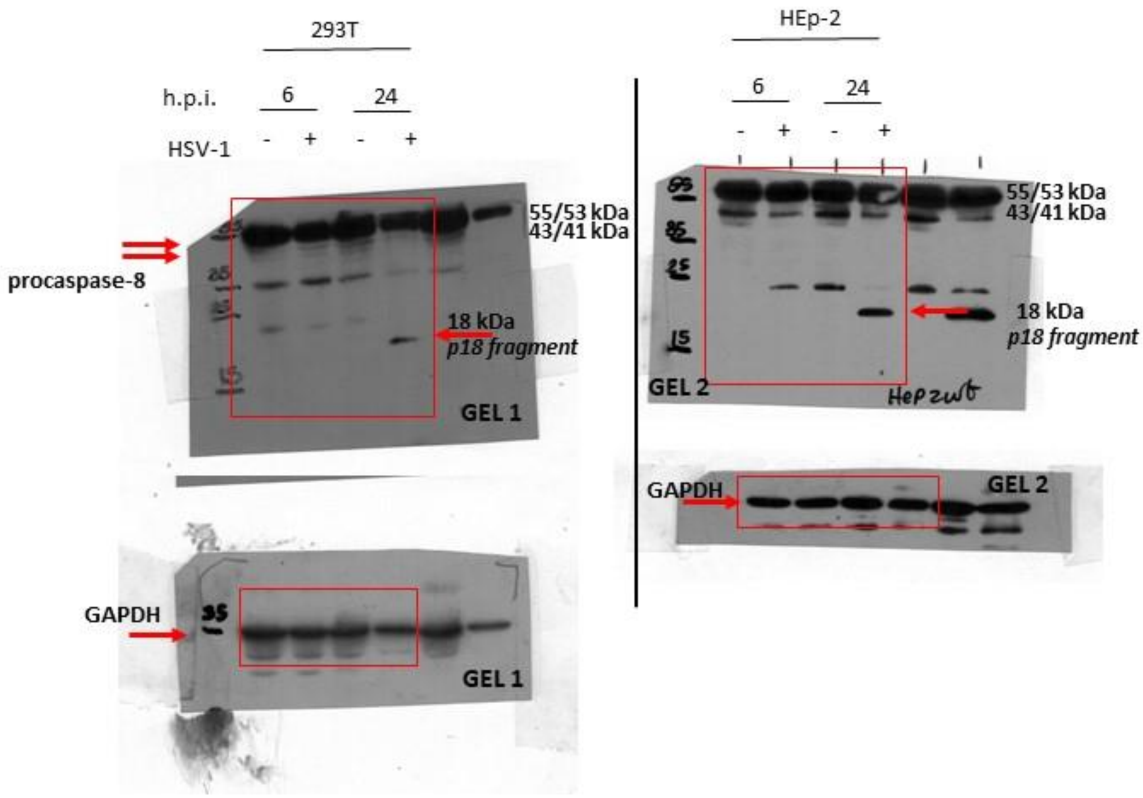

Figure S3. Analysis of apoptotic markers. d) analysis of caspase-8 in non-immune cells (293T and HEp-2) infected or not with HSV-1. The grouping blots are cropped from two different gels (GEL 1 and GEL 2,), as displayed in the figure. To improve the clarity and conciseness of the presentation, the figures were presented as cropping parts of the same gel first blotted with anti-caspase-8 antibody and then with anti-GAPDH. Arrowheads indicate bands corresponding to target proteins. The boxes indicate the lanes reported in the manuscript.

Supplementary figure S3.

Original image of Figure 3e

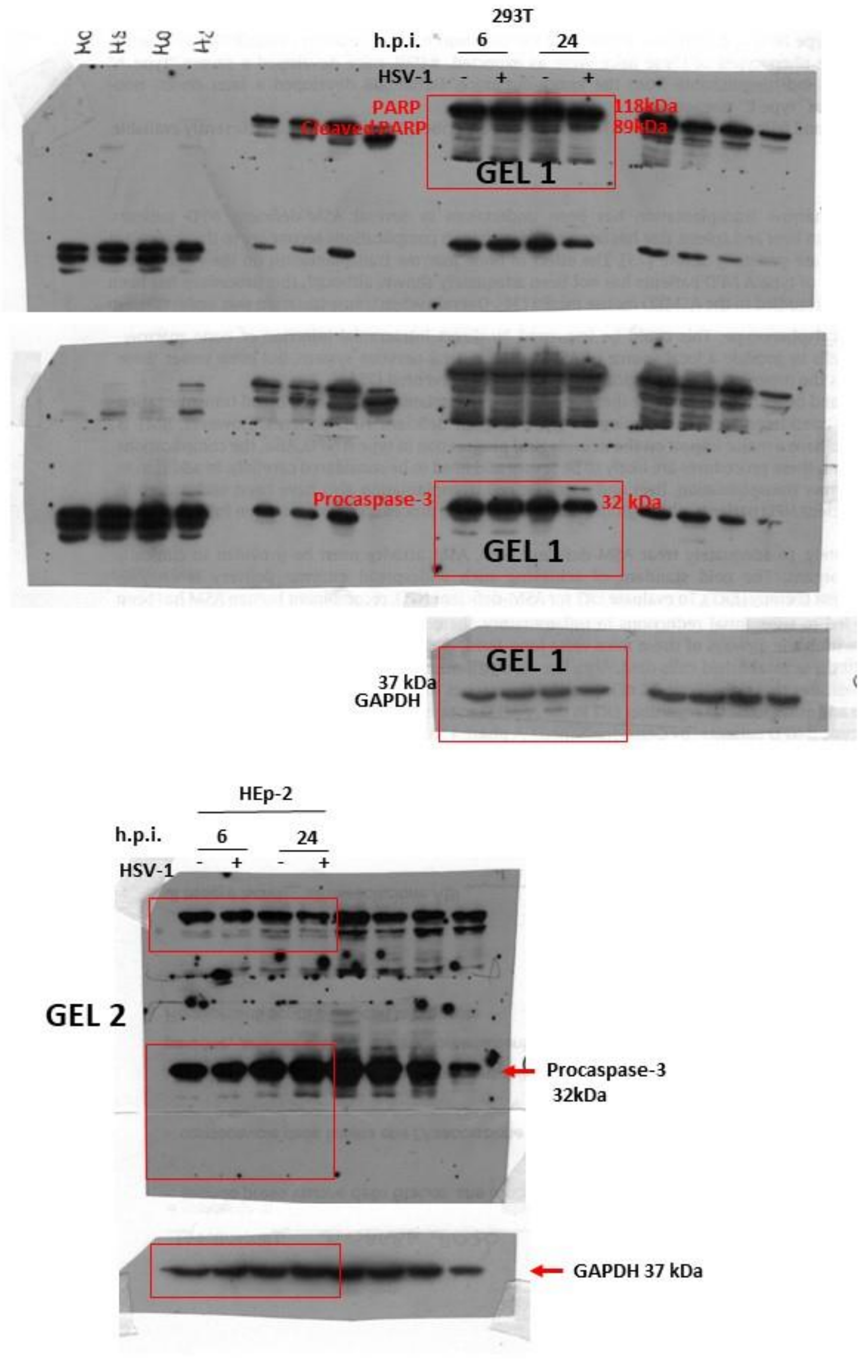

Figure S3. Analysis of apoptotic markers. e) analysis of apoptosis marker in non-immune cells (293T and HEp-2) infected or not with HSV-1. The grouping blots are cropped from two different gels (GEL 1 and GEL 2,), as displayed in the figure. the figures were presented as cropping parts of the same gel first blotted with anti-PARP antibody and then with anti-caspase-3 and anti-GAPDH. The boxes indicate the lanes reported in the manuscript. Arrowheads indicate bands corresponding to target proteins.
