# Supplementary material for: Direct cleavage of caspase-8 by herpes simplex virus 1 tegument protein US11
Source: Sci Rep. 2022 Jul 19;12:12317. doi: 10.1038/s41598-022-15942-9 (PMC9296525; doi:10.1038/s41598-022-15942-9)
Supplement: Supplementary file 4 — Supplementary Information 4. [file 41598_2022_15942_MOESM4_ESM.pdf]

Supplementary Information 4

Direct cleavage of Caspase-8 by Herpes Simplex Virus 1 Tegument Protein US11

Maria Musarra-Pizzo[1]*, Rosamaria Pennisi[1], Daniele Lombardo[2], Tania Velletri[3] and Maria Teresa Sciortino[1]*

[1]Department of Chemical, Biological, Pharmaceutical and Environmental Sciences, University of Messina, Messina, Italy, 98168, Europe.

[2]Division of Clinical and Molecular Hepatology, University Hospital 'G. Martino' of Messina, Messina, 98124, Italy

[3]IFOM-Cogentech Società Benefit srl; via Adamello 16, 20139 Milan, Italy-Local Unit: Scientific and Technological Park of Sicily- 95121 Catania, Italy.

*Corresponding authors: Maria Teresa Sciortino and Maria Musarra Pizzo

Supplementary figure S4.

Original image of Figure 4a

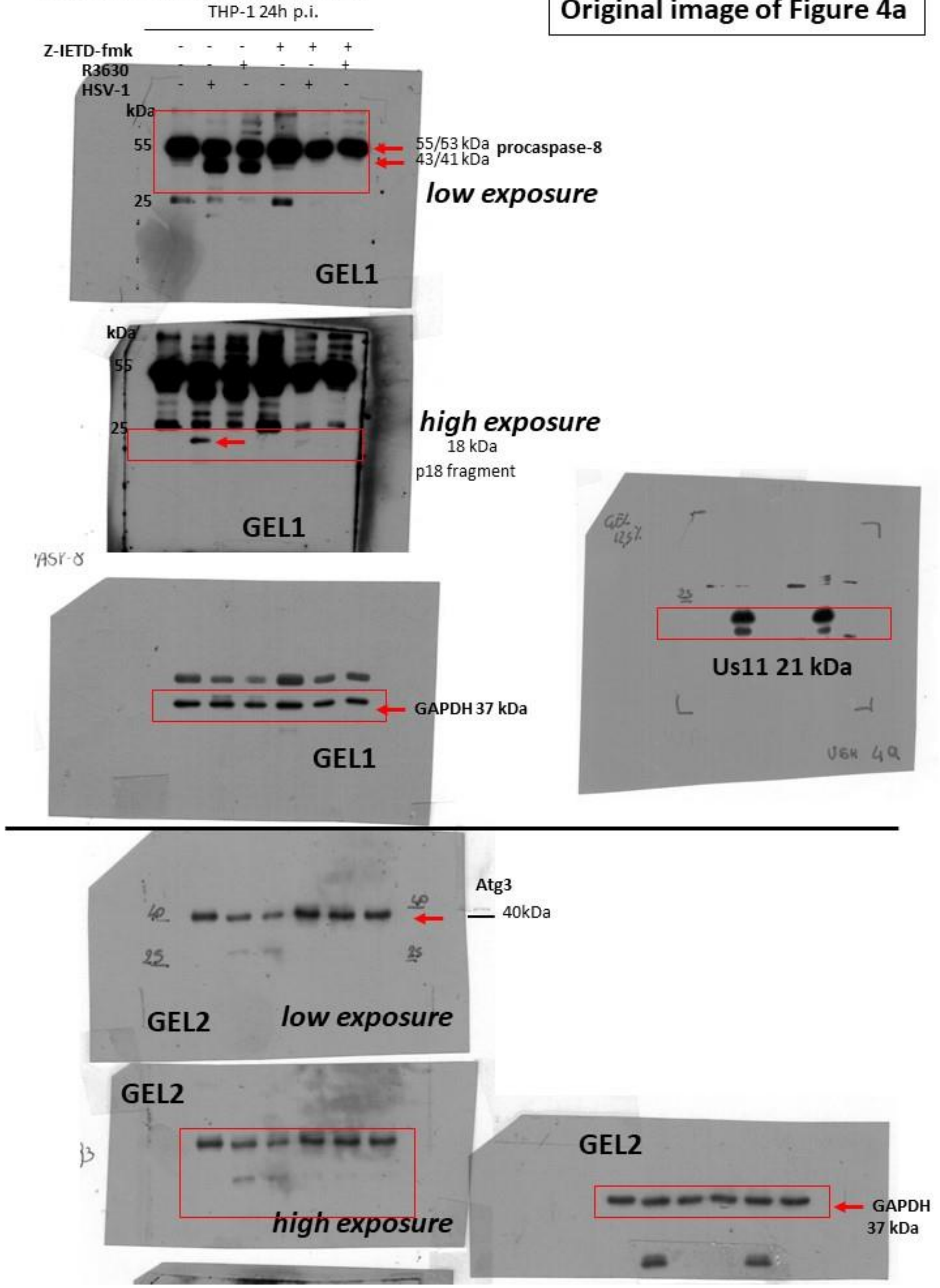

Fig. S4: Cleavage of Atg3 protein in infected or transfected THP-1 cells. (a)THP-1 cells were infected with HSV-1 or R3630 (ΔUs11/Us12), treated or not with caspase-8 inhibitor z-IETD-fmk (100 μM) and collected 24h and 48h p.i. Atg3 degradation was analyzed by immunoblotting. GAPDH was used as a loading control. The grouping blots are cropped from two different gels (GEL 1 and GEL 2,), as displayed in the figure. **GEL1:** Multiple exposures of cleavage of caspase 8 have been shown. To improve the clarity and conciseness of the presentation, the figure was presented as follow: procaspase-8 full length and p43/41 from low exposure; p18 fragment from high exposure. **GEL2:** the membrane was probed first with anti-ATG3 antibody and then with anti-GAPDH. The boxes indicate the lanes reported in the manuscript. Arrowheads indicate bands corresponding to target proteins

Supplementary figure S4.

Original image of Figure 4c

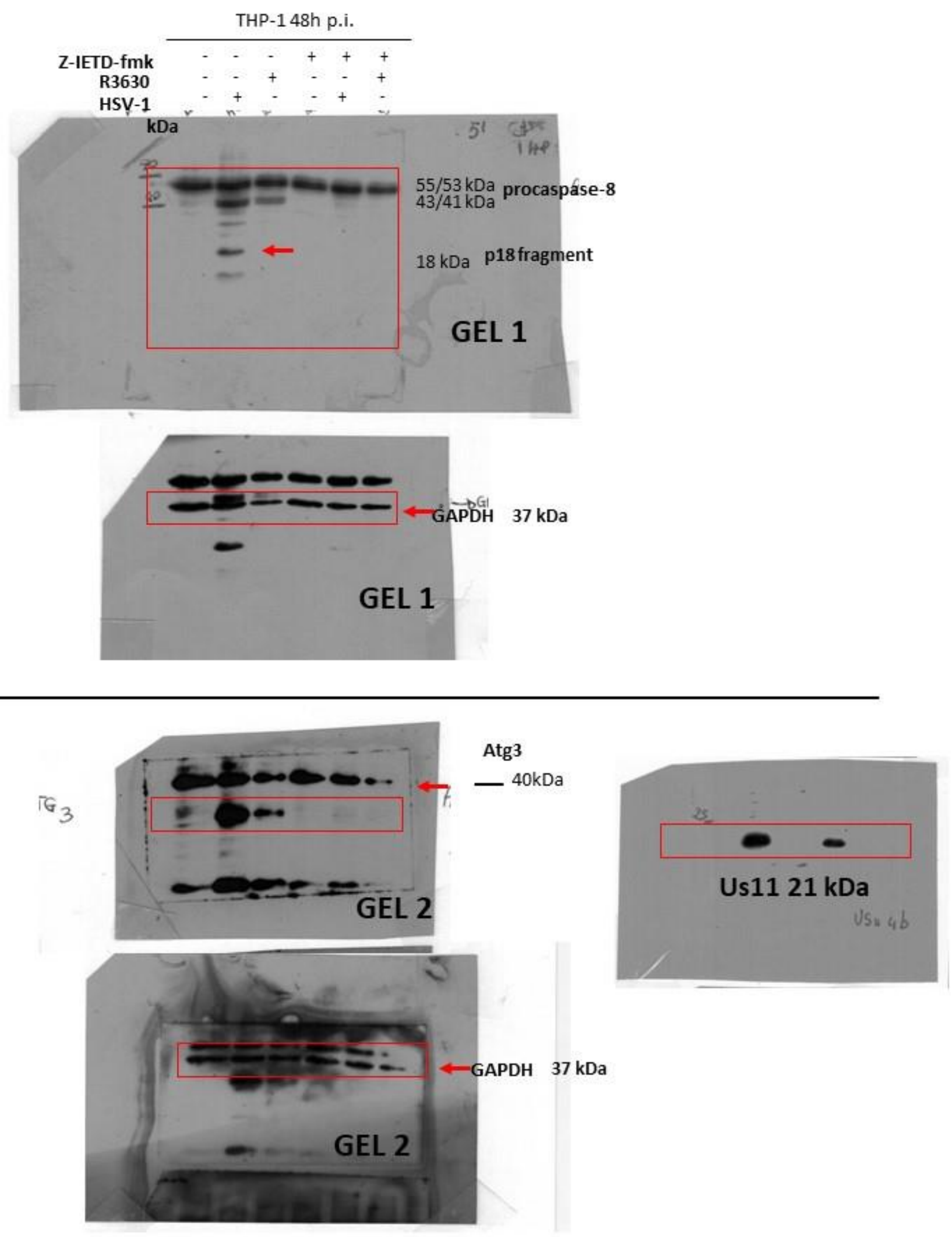

Fig.S4 Cleavage of Atg3 protein in infected or transfected THP-1 cells. (c)THP-1 cells were infected with HSV-1 or R3630 (ΔUs11/Us12), treated or not with caspase-8 inhibitor z-IETD-fmk (100 μM) and collected 48h p.i. Atg3 degradation was analyzed by immunoblotting. GAPDH was used as a loading control. The grouping blots are cropped from two different gels (GEL 1 and GEL 2,), as displayed in the figure. The boxes indicate the lanes reported in the manuscript. Arrowheads indicate bands corresponding to target proteins and the Atg3 fragment

Supplementary figure S4.

Original image of Figure 4e

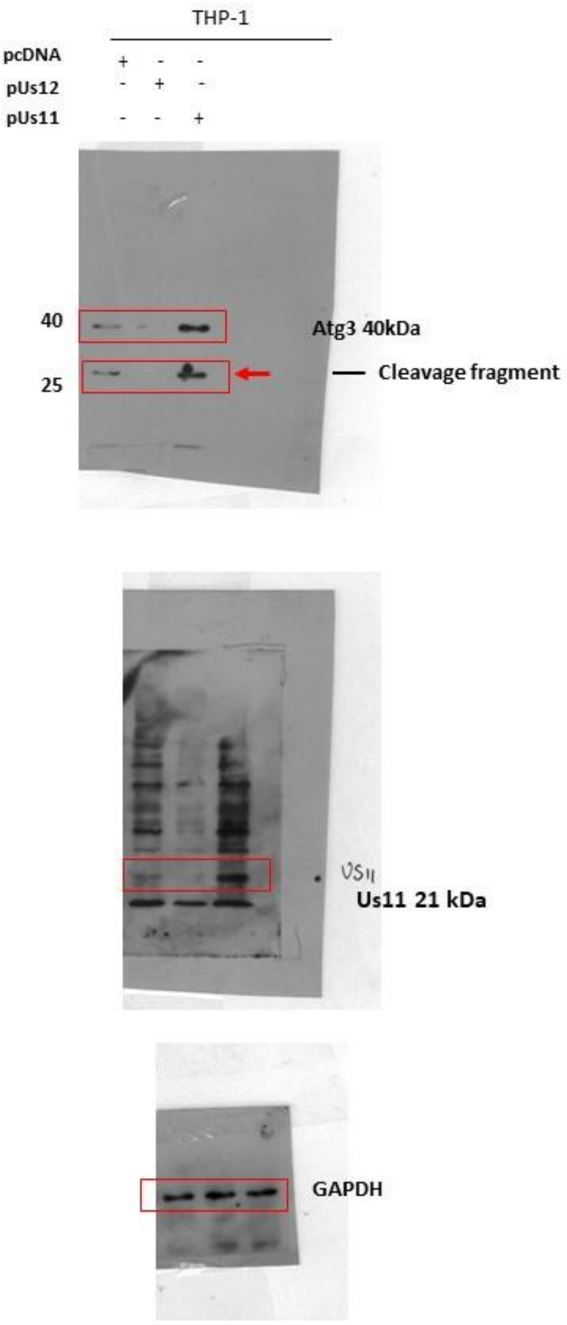

Fig.4: In vitro cleavage of Atg3 by caspase-8 during HSV-1 infection. (e)THP-1 cells were transfected with pUs11 and pUs12 plasmids and collected at 48h post-transfection. Atg3 degradation was analyzed by immunoblotting. GAPDH was used as a loading control. The boxes indicate the lanes reported in the manuscript. Arrowheads indicate bands corresponding to target proteins and the Atg3 fragment.
