# Supplementary material for: Direct cleavage of caspase-8 by herpes simplex virus 1 tegument protein US11
Source: Sci Rep. 2022 Jul 19;12:12317. doi: 10.1038/s41598-022-15942-9 (PMC9296525; doi:10.1038/s41598-022-15942-9)
Supplement: Supplementary file 5 — Supplementary Information 5. [file 41598_2022_15942_MOESM5_ESM.pdf]

Supplementary Information 5

Direct cleavage of Caspase-8 by Herpes Simplex Virus 1 Tegument Protein US11

Maria Musarra-Pizzo[1]*, Rosamaria Pennisi[1], Daniele Lombardo[2], Tania Velletri[3] and Maria Teresa Sciortino[1]*

[1]Department of Chemical, Biological, Pharmaceutical and Environmental Sciences, University of Messina, Messina, Italy, 98168, Europe.

[2]Division of Clinical and Molecular Hepatology, University Hospital 'G. Martino' of Messina, Messina, 98124, Italy

[3]IFOM-Cogentech Società Benefit srl; via Adamello 16, 20139 Milan, Italy-Local Unit: Scientific and Technological Park of Sicily- 95121 Catania, Italy.

*Corresponding authors: Maria Teresa Sciortino and Maria Musarra Pizzo

Supplementary figure S5.

Original image of Figure 5a

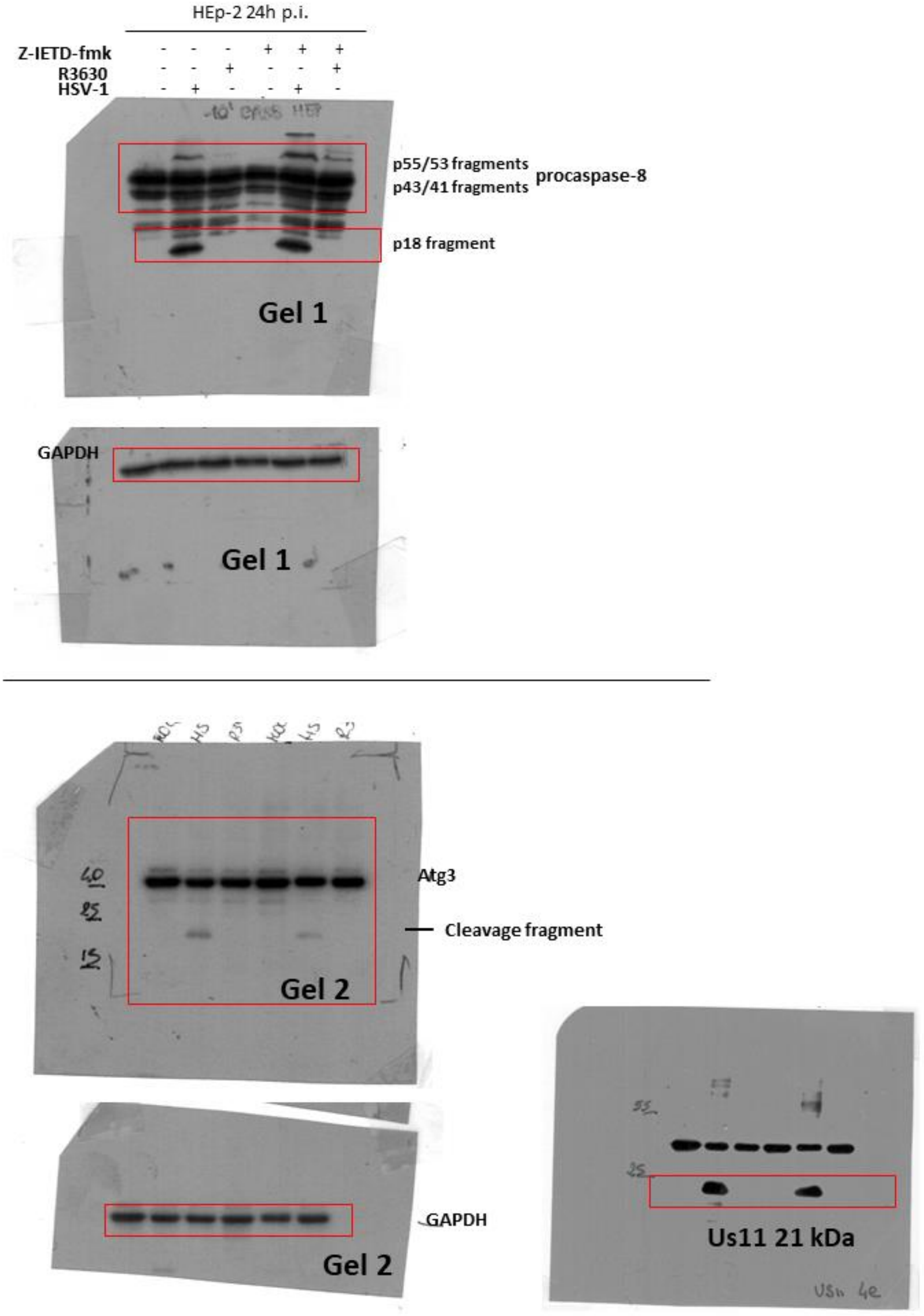

Fig. S5 Cleavage of Atg3 protein in HEp-2 infected cells. (a) HEp-2 cells were infected with HSV-1 or R3630 (ΔUs11/Us12), treated or not with caspase-8 inhibitor z-IETD-fmk (100 µM) and collected 24h p.i. Atg3 degradation was analyzed by immunoblotting. GAPDH was used as a loading control. Arrows denote the cleavage fragments. The grouping blots are cropped from two different gels (GEL 1 and GEL 2,), as displayed in the figure. To improve the clarity and conciseness of the presentation, the figure was presented as cropping parts of the same gel first blotted with anti-caspase-8 antibody and then with anti-GAPDH. The boxes indicate the lanes reported in the manuscript.

Supplementary figure S5.

Original image of Figure 5c

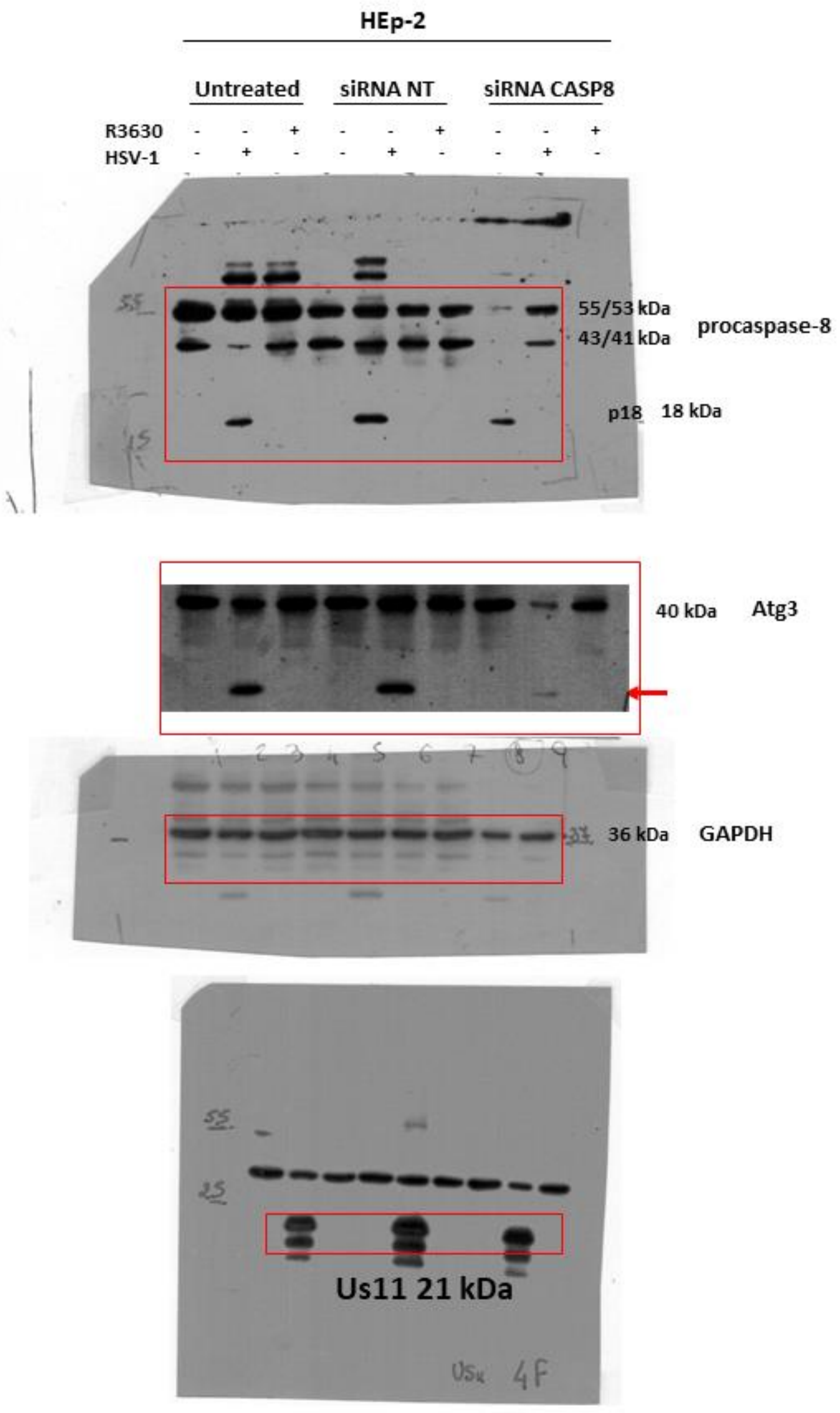

Fig. S5 Cleavage of Atg3 protein in HEp-2 infected cells. (c) Knockdown of Caspase-8 was performed using a pool of siRNAs. HEp-2 (2.5 X 10 5 cell/well) were seeded onto 6 well plates for 24 h. Then, 300 nM of each siRNAs targeting different region of caspase-8 (siRNA CASP8) or negative control siRNA (siRNA NT) were transfected on HEp-2 cells. Untreated transfected cells were set as control. Then, 48h post transfection the cells were infected for further 24h with HSV-1 or R3630 (ΔUs11/Us12) or not and collected for immunoblotting. Arrows denote the cleavage fragments. The boxes indicate the lanes reported in the manuscript

Supplementary figure S5.

Original image of Figure 5e

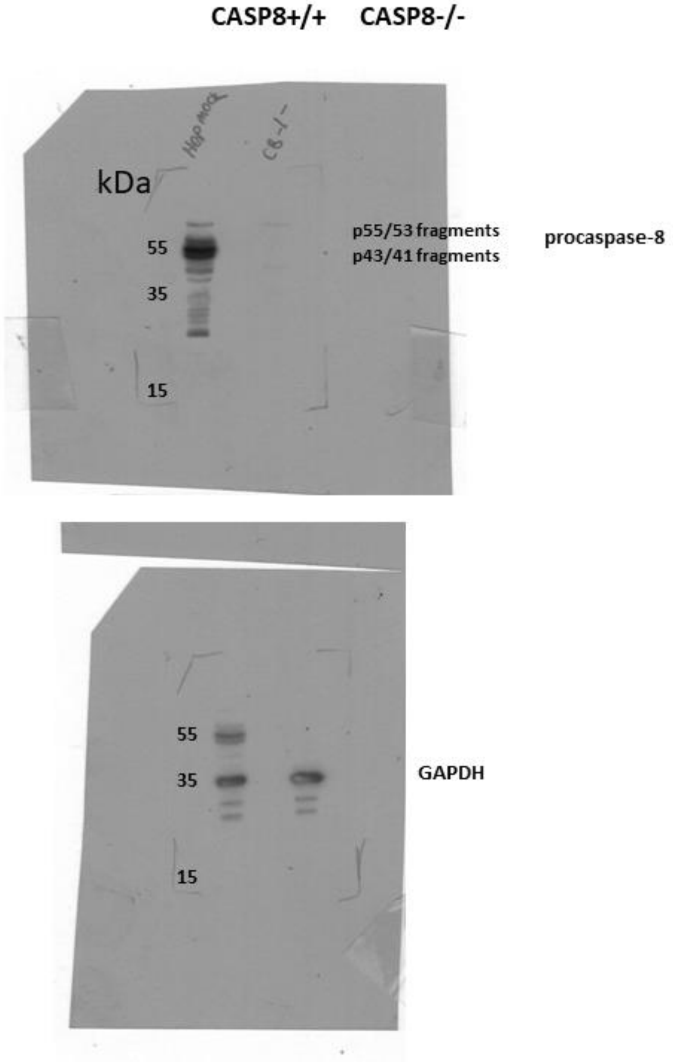

Fig. S5 Cleavage of Atg3 protein in HEp-2 infected cells. (e) Immunoblot analyses of lysates of HEp-2 Caspase-8 knocked-out cells (CASP8-\-) and wild type (CASP8+\+) for accumulation of knocked-out gene.

Supplementary figure S5.

Original image of Figure 5f

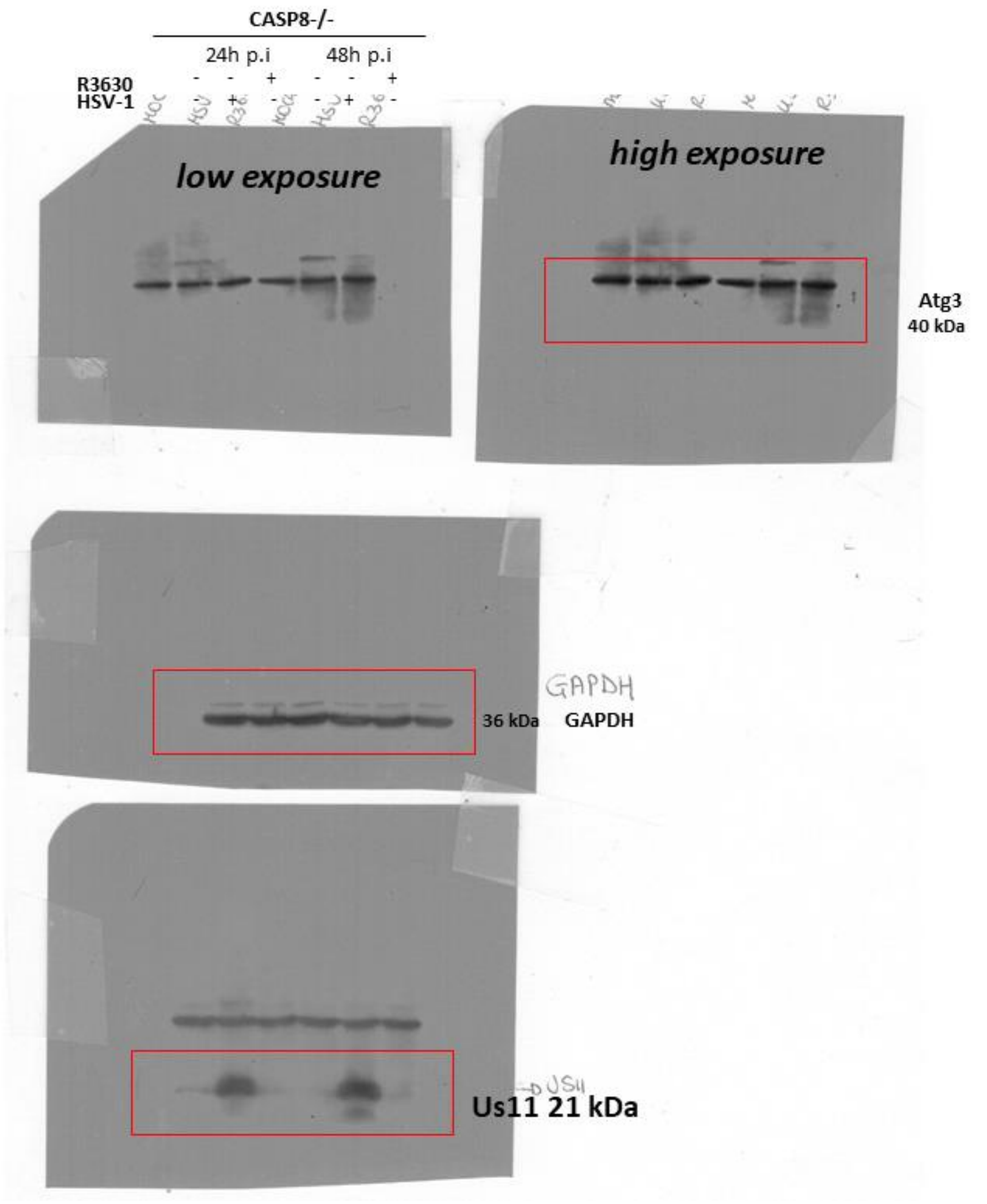

Fig. S5 Cleavage of Atg3 protein in HEp-2 infected cells. (f) CASP 8-/- cells were infected with HSV-1 or R3630 (ΔUs11/Us12), and collected 24h and 48h p.i. Atg3 degradation was analyzed by immunoblotting. GAPDH was used as a loading control. Multiple exposures of ATG3 have been shown. The boxes indicate the lanes reported in the manuscript.
