# Supplementary material for: Direct cleavage of caspase-8 by herpes simplex virus 1 tegument protein US11
Source: Sci Rep. 2022 Jul 19;12:12317. doi: 10.1038/s41598-022-15942-9 (PMC9296525; doi:10.1038/s41598-022-15942-9)
Supplement: Supplementary file 6 — Supplementary Information 6. [file 41598_2022_15942_MOESM6_ESM.pdf]

Supplementary Information 6

Direct cleavage of Caspase-8 by Herpes Simplex Virus 1 Tegument Protein US11

Maria Musarra-Pizzo[1]*, Rosamaria Pennisi[1], Daniele Lombardo[2], Tania Velletri[3] and Maria Teresa Sciortino[1]*

[1]Department of Chemical, Biological, Pharmaceutical and Environmental Sciences, University of Messina, Messina, Italy, 98168, Europe.

[2]Division of Clinical and Molecular Hepatology, University Hospital ‘G. Martino’ of Messina, Messina, 98124, Italy

[3]IFOM-Cogentech Società Benefit srl; via Adamello 16, 20139 Milan, Italy-Local Unit: Scientific and Technological Park of Sicily- 95121 Catania, Italy.

*Corresponding authors: Maria Teresa Sciortino and Maria Musarra Pizzo

Supplementary figure S6.

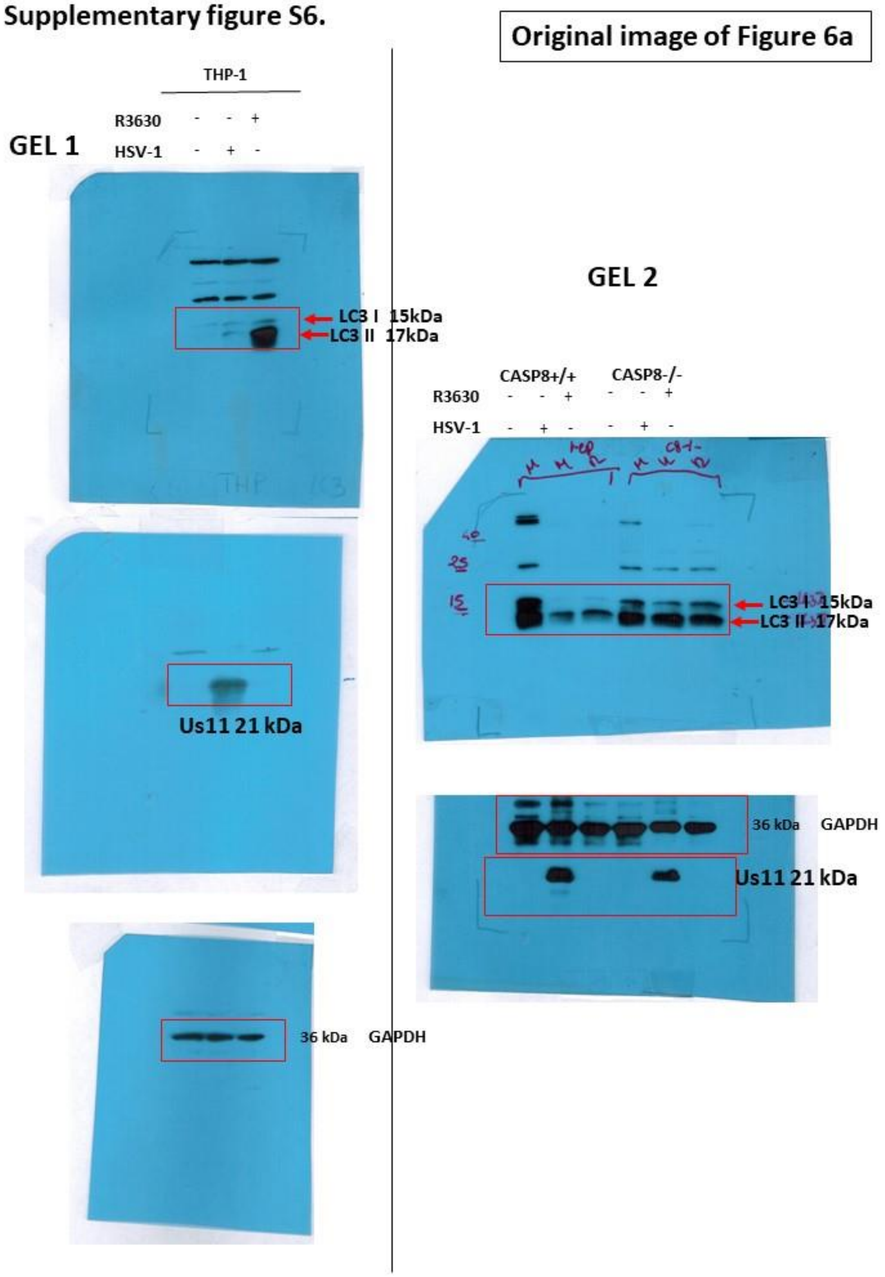

Figure S6: Lipidation of LC3 in THP-1, CASP8+/+ and CASP8-/- cells: a) THP-1 cells were infected at 50 MOI with HSV-1 and R3630 and collected 48h p.i. CASP8+/+ and CASP8-/- cells were infected at 10 MOI with HSV-1 and R3630 and collected 24h p.i. Cells were then lysed and the LC3 expression was detected by immunoblotting. GAPDH was used as a loading control. The LC3-II/LC3-I ratios of HSV-1 and R3630 infected samples was normalised to the LC3-II/LC3-I ratio of control-uninfected cells. The grouping blots are cropped from two different gels (GEL 1 and GEL 2,), as displayed in the figure. The boxes indicate the lanes reported in the manuscript. Arrowheads indicate bands corresponding to target proteins.
