# Supplementary material for: Direct cleavage of caspase-8 by herpes simplex virus 1 tegument protein US11
Source: Sci Rep. 2022 Jul 19;12:12317. doi: 10.1038/s41598-022-15942-9 (PMC9296525; doi:10.1038/s41598-022-15942-9)
Supplement: Supplementary file 7 — Supplementary Information 7. [file 41598_2022_15942_MOESM7_ESM.pdf]

Supplementary Information 7

Direct cleavage of Caspase-8 by Herpes Simplex Virus 1 Tegument Protein US11

Maria Musarra-Pizzo[1]*, Rosamaria Pennisi[1], Daniele Lombardo[2], Tania Velletri[3] and Maria Teresa Sciortino[1]*

[1]Department of Chemical, Biological, Pharmaceutical and Environmental Sciences, University of Messina, Messina, Italy, 98168, Europe.

[2]Division of Clinical and Molecular Hepatology, University Hospital 'G. Martino' of Messina, Messina, 98124, Italy

[3]IFOM-Cogentech Società Benefit srl; via Adamello 16, 20139 Milan, Italy-Local Unit: Scientific and Technological Park of Sicily- 95121 Catania, Italy.

*Corresponding authors: Maria Teresa Sciortino and Maria Musarra Pizzo

Supplementary figure S7.

Original image of Figure 7a

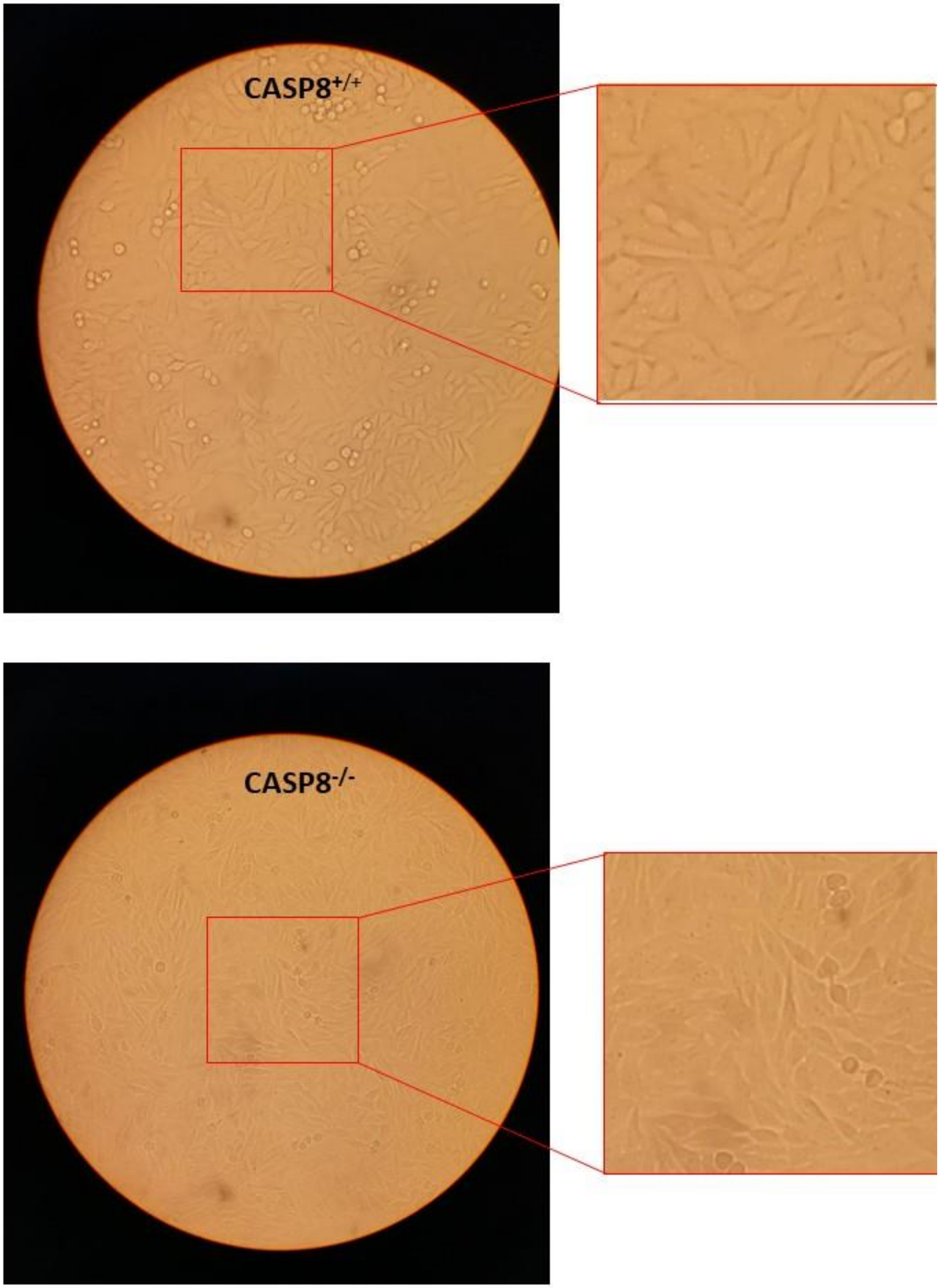

Supplementary figure S7.

Original image of Figure 7a

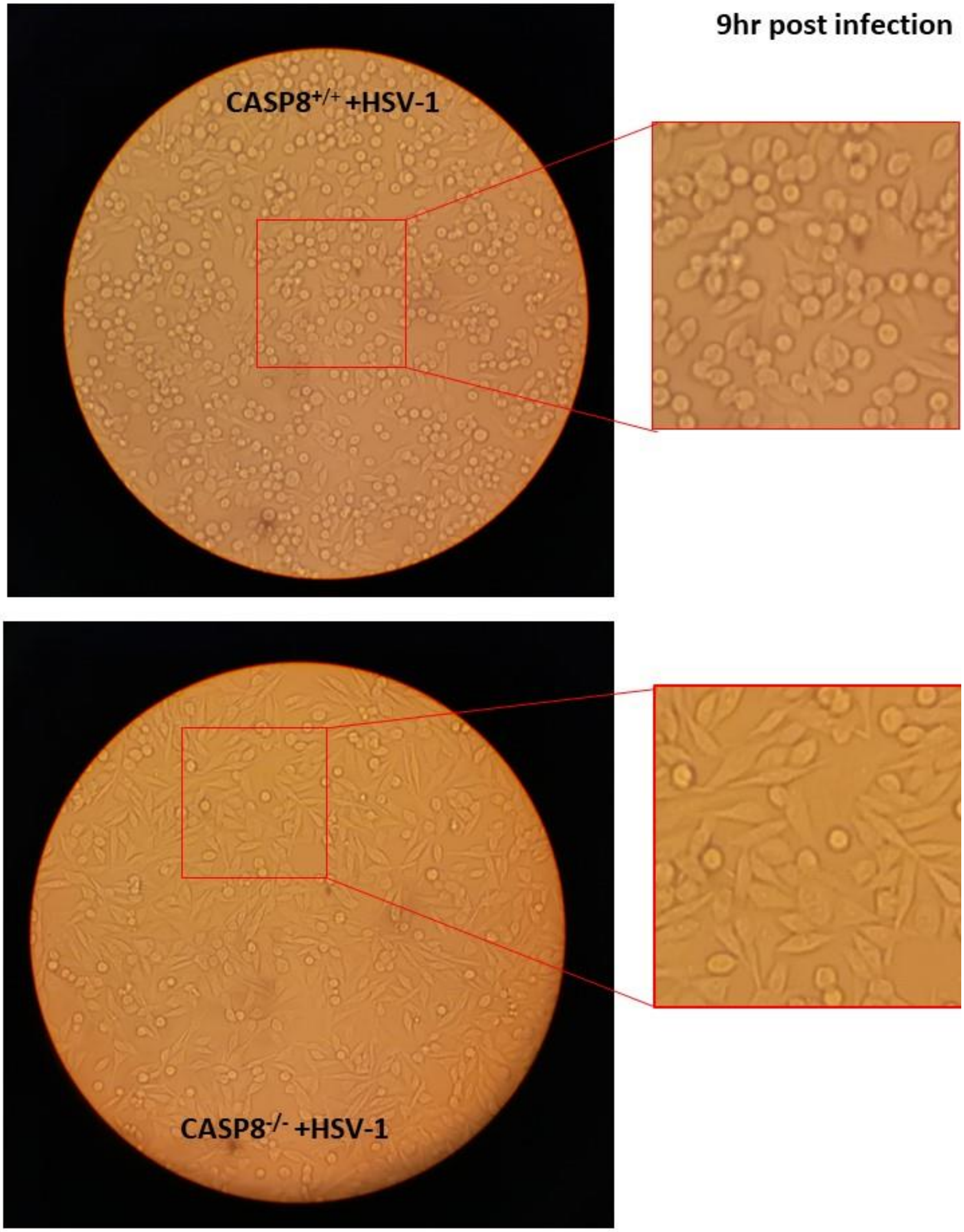

Supplementary figure S7.

Original image of Figure 7a

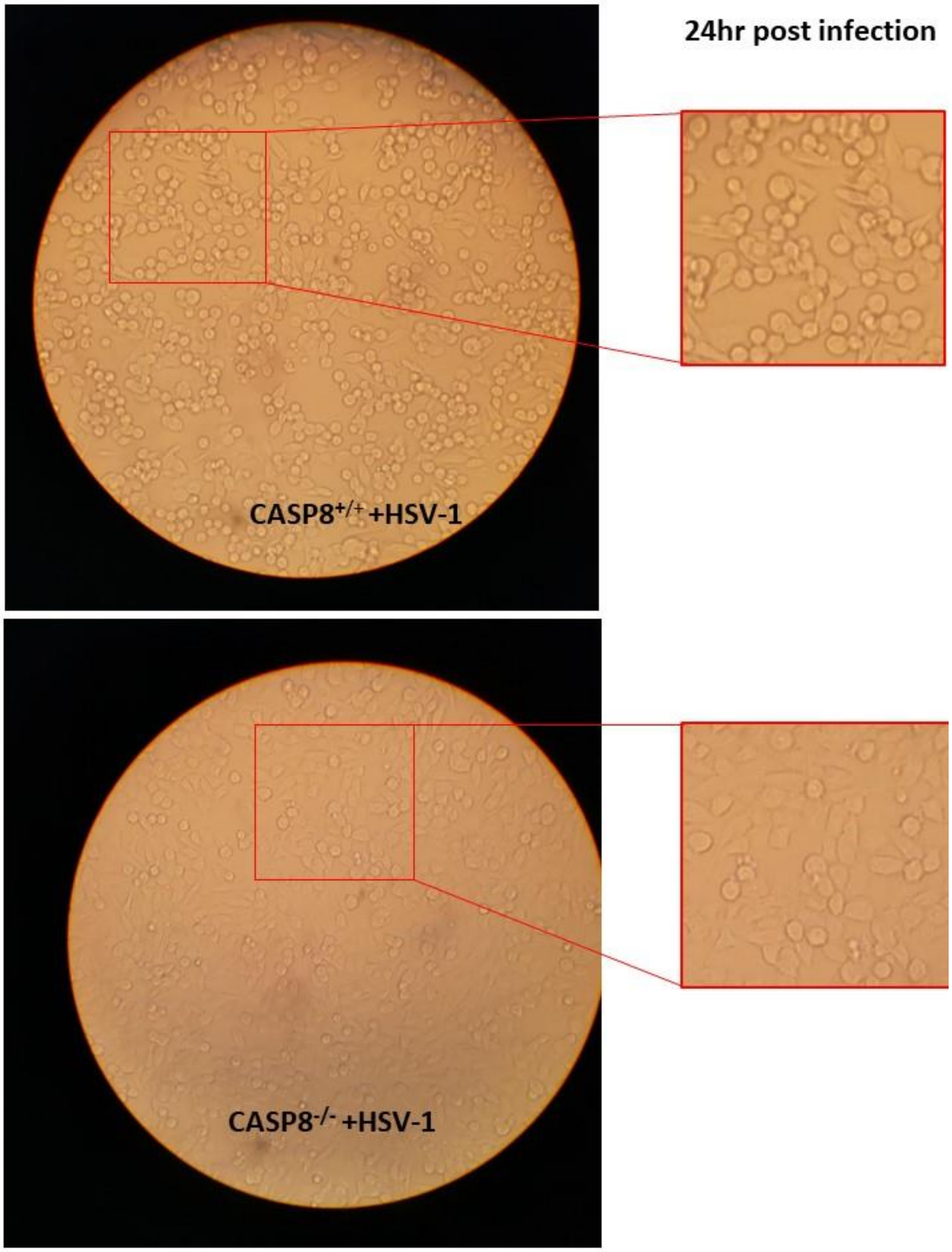

Figure S7. Comparison of HSV-1 replication efficiency in $CASP8^{+\backslash+}$ and $CASP8^{-\backslash-}$ cells. a) Caspase-8 deficient ($CASP8^{-\backslash-}$) and the wild type ($CASP8^{+\backslash+}$) cell lines were infected with HSV-1 and collected at 9h and 24h post infection. The cytopathic effect was observed under an inverted light microscopy (magnification x10). The original images of the microscope analysis were reported and the red boxes show the detail presented in the manuscript.

Supplementary figure S7.

Original image of Figure 7e

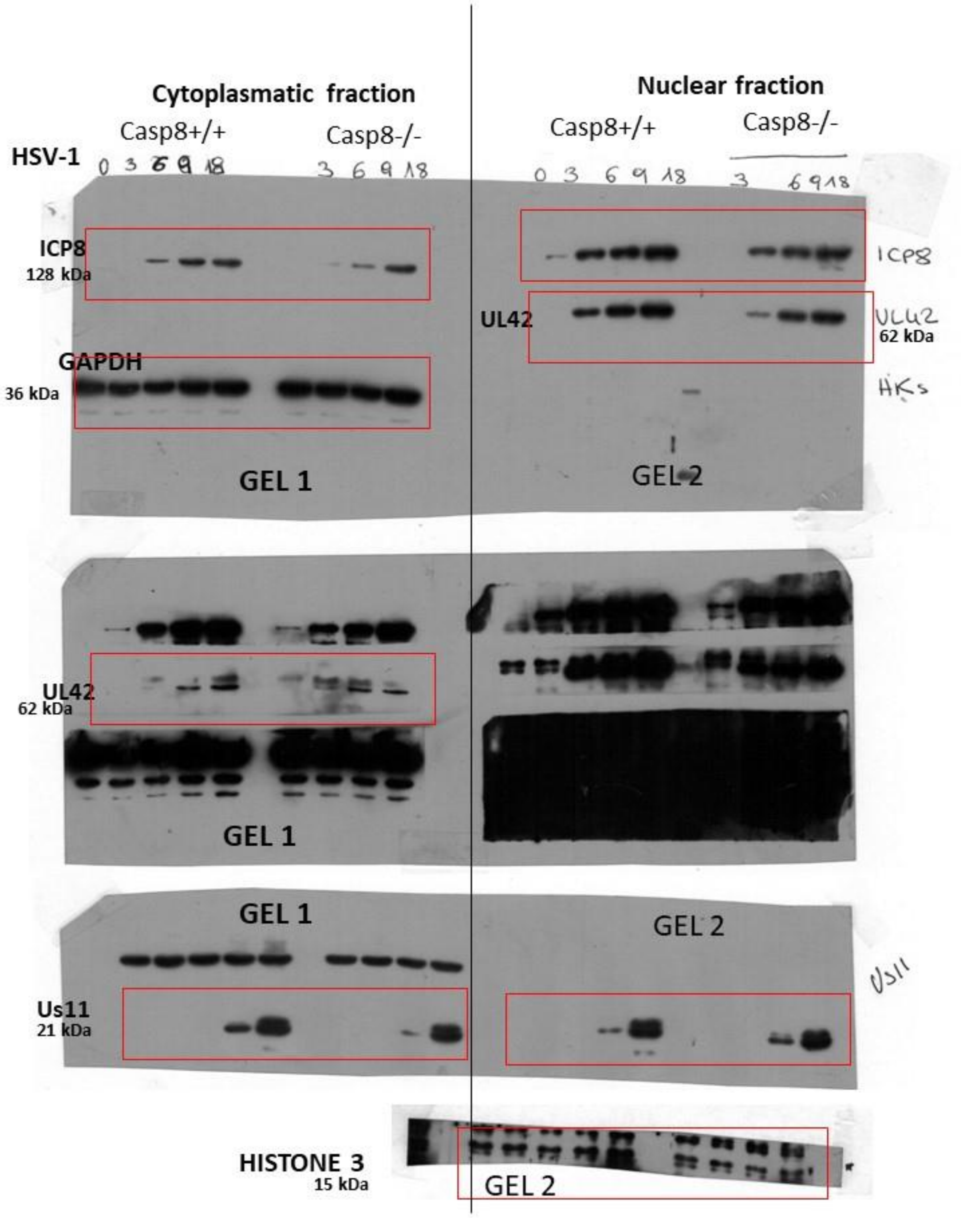

Figure S7. Comparison of HSV-1 replication efficiency in CASP8$^{+\backslash+}$ and CASP8$^{-\backslash-}$ cells. (e) Caspase-8 deficient (CASP8$^{-\backslash-}$) and the wild type (CASP8$^{+\backslash+}$) cell lines were infected with HSV-1 and collected at several time p.i. equal amount of cytoplasmic and nuclear proteins were resolved by SDS-PAGE and the membranes were probed with antibodies directed against the viral protein ICP8 (β), UL42 (β) and Us11 (γ2). GAPDH and Histone 3 were used as a loading control. The grouping blots are cropped from two different gels (GEL 1 and GEL 2,), as displayed in the figure, and exposed simultaneously. The red boxes indicate the lanes reported in the manuscript.
